# 3′-UTR Polymorphisms in the Vascular Endothelial Growth Factor Gene (VEGF) Contribute to Susceptibility to Recurrent Pregnancy Loss (RPL)

**DOI:** 10.3390/ijms20133319

**Published:** 2019-07-05

**Authors:** Hui Jeong An, Ji Hyang Kim, Eun Hee Ahn, Young Ran Kim, Jung Oh Kim, Han Sung Park, Chang Soo Ryu, Eun-Gyo Kim, Sung Hwan Cho, Woo Sik Lee, Nam Keun Kim

**Affiliations:** 1 Department of Biomedical Science, College of Life Science, CHA University, Seongnam 13488, Korea; 2 Department of Obstetrics and Gynecology, CHA Bundang Medical Center, CHA University, Seongnam 13496, Korea; 3 Fertility Center of CHA Gangnam Medical Center, CHA University, Seoul 06135, Korea

**Keywords:** recurrent pregnancy loss, polymorphism, *VEGF* 3′-untranslated region, haplotypes, single nucleotide polymorphisms, biomarkers

## Abstract

Numerous studies have examined the genetic association of vascular endothelial growth factor (VEGF) single nucleotide polymorphisms (SNPs) with recurrent pregnancy loss (RPL). However, of the four known SNPs in the 3′-untranslated region (3′-UTR) of *VEGF*, three SNPs—namely rs3025040 (1451C>T), rs10434 (1612G>A), and rs3025053 (1725G>A)—remain poorly characterized with regard to RPL. Herein, we evaluated the association between these three SNPs in the *VEGF* 3′-UTR and RPL susceptibility. We analyzed *VEGF* 3′-UTR gene variants in with and without RPL using TaqMan allelic discrimination. There were significant differences in the genotype frequencies of 1612G>A (GA: adjusted odds ratio (AOR), 0.652; 95% confidence interval (CI), 0.447–0.951; *p* = 0.026) and 1725G>A (GA: AOR, 0.503; 95% CI, 0.229–0.848; *p* = 0.010) in RPL patients vs. controls. Our results indicate that the 1612G>A and 1725G>A polymorphisms in the 3′-UTR of *VEGF* are associated with RPL susceptibility in Korean women. These data suggest that *VEGF* 3′-UTR polymorphisms may be utilized as biomarkers for the detection of RPL risk and prevention.

## 1. Introduction

Recurrent pregnancy loss (RPL) is commonly defined as more than two consecutive miscarriages prior to 20 weeks [1], although the American Society for Reproductive Medicine has expanded its definition to include two consecutive losses [2]. RPL is experienced by approximately 2–4% of reproductive-age women, a considerable number, and is morally devastating for couples who wish to become pregnant, RPL is also often disappointing for clinicians.

Critically, no diagnostic factors have been identified that distinguish RPL patients that have suffered different numbers of pregnancy losses [3]. RPL etiology remains unclear [4], although the causes of RPL are now known to include anatomic factors, hormonal, and metabolic factors, and antiphospholipid syndrome. Vascular endothelial growth factor-A (*VEGF*-A), belonging to the same protein family as *VEGF* proteins B, C, and D, as well as placenta growth factor, is a glycoprotein that exists as a disulfide-bonded dimer. The *VEGF-A* gene encompasses 14 kb on human chromosome 6 and has eight exons [5]. 

Of the *VEGF* family members, *VEGF*-A (also known as *VEGF*) is the principal inducer of angiogenesis and, among its many roles, is critical for the stimulation of trophoblast proliferation, embryonic vasculature development, and maternal and fetal blood cell growth during early pregnancy [6,7,8,9,10]. The *VEGF* protein family includes *VEGF*-A (i.e., *VEGF*), *VEGF*-B, *VEGF*-C, and *VEGF*-D, all of which are important modifiers of angiogenesis. *VEGF* is particularly important for both angiogenesis, a process that contributes to the formation of the vascular system in developing embryos (i.e., vasculogenesis), and the implantation of embryos into the placental wall. Furthermore, because *VEGF* is required to initiate angiogenesis, it contributes to atherothrombotic vascular disease progression including ischemic stroke. Various lines of evidence point to the importance of *VEGF* and its receptors in embryonic and vascular angiogenesis. Angiogenesis occurs normally in female reproductive organs; therefore, when nonpregnant, cycling female mice are treated with angiogenesis inhibitor AGM-1470, endometrial maturation and corpora lutea development are also inhibited. In pregnant mice, this inhibition of *VEGF* interferes with many critical processes, resulting in embryonic growth failure and thus suggesting a critical role of this protein during pregnancy [11]. 

Numerous genetic association studies have examined the possible link between single nucleotide polymorphisms (SNPs) in *VEGF* and RPL susceptibility [12]. A recent meta-analysis, for example, indicated that polymorphisms at rs1570360, rs3025039, rs2010963, and rs3025020 are significantly associated with RPL susceptibility. However, despite the interest in this gene, only a limited number of *VEGF* loci have been carefully studied for their role in RPL. The *VEGF* 3′-untranslated region (3′-UTR) contains four known SNPs (rs3025039, rs3025040, rs10434, rs3025053). However, only rs3025039 has been characterized in relation to RPL. Therefore, herein we investigated the association of SNPs rs3025040 (1451C>T), rs10434 (1612G>A), and rs3025053 (1725G>A) in the 3′-UTR of *VEGF* with RPL risk.

## 2. Results

### Studied VEGF 3′-UTR SNPs Are in Complete Hardy–Weinberg Equilibrium

Table 1 shows the baseline characteristics of control subjects and RPL patients. In the RPL group, the averages for gestational age and instances of pregnancy loss were 7.38 ± 1.92 weeks and 3.29 ± 1.85 losses, respectively. In the control group, the averages for gestational age and number of live births were 39.26 ± 1.66 weeks and 1.72 ± 0.72 births, respectively. There is a significant difference in pregnancy loss and gestational age when comparing controls and patients. Factors related with pregnancy include mean gestational age (weeks), PLT (103/μL), Hct (%), LH (mIU/mL), E2 (pg/mL) with *p*-value less than 0.05 significant to RPL. Other factors were not significant with RPL.

Table 2 shows the *VEGF* 1451C>T, 1612G>A, and 1725G>A genotype frequencies in control and RPL patient groups. The frequencies of 1612G>A polymorphisms (75.4% GG, 21.7% GA, 2.9% AA) were significantly different between RPL patients and controls (66.1% GG, 28.8% GA, 5.1% AA). We further found that the *VEGF* 1612A allele decreased RPL risk by 0.654-fold (AOR, 0.654; 95% CI, 0.481–0.891; *p* = 0.007). Similarly, 1725G>A polymorphism frequencies (92.1% GG, 7.9% GA, 0.0% AA) in RPL patients also differed from those in controls (84.3% GG, 14.4% GA, 1.3% AA). In this case, the *VEGF* 1725A allele reduced RPL risk by 0.446-fold (AOR, 0.446; 95% CI, 0.273–0.726; *p* = 0.001). Conversely, no significant associations were observed between *VEGF* 1451C>T and RPL susceptibility. From these results, we propose that the *VEGF* 1612G>A, and 1725G>A SNPs may predispose individuals to RPL.

We next compared genotype frequencies with regard to the instances of pregnancy loss. In subjects with two or more pregnancy losses, the frequencies of *VEGF* 1612G>A and *VEGF* 1725G>A genotypes were significantly lower than in controls: *VEGF* 1612G>A (GG vs. GA: AOR, 0.476; 95% CI, 0.293–0.774 and GG vs. GA+AA: AOR, 0.446; 95% CI, 0.280–0.710) and *VEGF* 1725G>A (GG vs. GA: AOR, 0.413; 95% CI, 0.207–0.825 and GG vs. GA+AA: AOR, 0.381; 95% CI, 0.192–0.755). 

No significant associations were found between the haplotype frequencies of the three *VEGF* SNPs and elevated RPL susceptibility, although some haplotypes were associated with decreased RPL susceptibility (Table 3). Specifically, the 1451C-1612A-1725A haplotype decreased RPL risk 0.507-fold (OR, 0.507; 95% CI, 0.303–0.839; *p* = 0.011), the 1451C-1612A haplotype lowered RPL risk 0.712-fold (OR, 0.712; 95% CI, 0.512–0.979; *p* = 0.040), the 1451C-1725A haplotype decreased RPL risk 0.457-fold (OR, 0.457; 95% CI, 0.279–0.746; *p* = 0.002), and the 1612A-1725A haplotype reduced RPL risk 0.490-fold (OR, 0.490; 95% CI, 0.294–0.817; *p* = 0.007). Finally, we performed a combined genotype analysis for the *VEGF* 3′-UTR polymorphisms analyzed in this study (1451C>T/1612G>A, 1451C>T/1725G>A, and 1612G>A/1725G>A), the results of which are shown in Table 4. We found that two SNP combinations were associated with RPL: 1451CC/1725GA (AOR, 0.510; 95% CI, 0.290–0.898; *p* = 0.020), and 1612GA/1725GA (AOR, 0.472; 95% CI, 0.258–0.866; *p* = 0.015).

We conducted statistical analyses of RPL incidence with respect to interaction with the following environmental factors: BMI, PLT, PT, aPTT, and levels of Hct, FSH, LH, and E2. Among these factors, only an FSH level ≤7.53 mIU/mL was significant and only in the *VEGF* 1451CT+TT genotype (AOR, 4.635; 95% CI, 2.093–10.263) (Table 5). Statistically significant clinical variables in RPL patients, stratified by *VEGF* polymorphisms, were as follows: BUN (mg/dL), *VEGF* 1725 G>A GG type (9.81 ± 2.77, *p* = 0.050); Cr (mg/dL), *VEGF* 1725G>A G allele (0.72 ± 0.12, *p* = 0.034); total cholesterol (mg/dL), *VEGF* 1725G>A G allele (187.80 ± 49.42, *p* = 0.036); FBS (mg/dL), *VEGF* 1725G>A G allele (95.19 ± 17.15, *p* = 0.017); Hct (%), *VEGF* 1451C>T CC type (36.88 ± 3.89, *p* = 0.032); HDL (mg/dL), *VEGF* 1451C>T CC type (53.72 ± 13.62, *p* = 0.048); LH (mIU/mL), *VEGF* 1612G>A GG type (4.91 ± 6.26, *p* = 0.001), Gallele (4.77 ± 5.48, *p* = 0.001), *VEGF* 1725G>A GG type (4.79 ± 5.69, *P* = 0.030); PT (sec), *VEGF* 1612G>A GG type (11.56 ± 1.42, *p* = 0.003), G allele (11.50 ± 1.31, *p* = 0.001), *VEGF* 1725G>A GG type (11.49 ± 1.34, *p* = 0.035) (Table 6).

Finally, in accordance with the quintiles of clinical variables, multiple linear regression analyses of clinical variables in Korean RPL patients identified the following variables as being statistically significant: homocysteine (Hcy), *VEGF* 1612G>A AA type (*p* = 0.048), recessive (GG+GA vs. AA) (P = 0.049); BUN, *VEGF* 1725G>A GA type (*p* = 0.037), dominant (GG vs. GA+AA) (*p* = 0.037); total cholesterol, *VEGF* 1612G>A GA type (*p* = 0.032); Hct, *VEGF* 1451C>T CT type (*p* = 0.014), dominant (CC vs. CT+TT) (*p* = 0.014); PLT, *VEGF* 1451C>T CT type (*P* = 0.005), dominant (CC vs. CT+TT) (*p* = 0.006); aPTT, *VEGF* 1612G>A GA type (*p* = 0.044) (Table 7).

## 3. Discussion

RPL is a complicated disease, and various genetic polymorphisms contribute to RPL risk [13]. Herein, we investigated the association between SNPs in the 3′-UTR of *VEGF* and RPL susceptibility in a cohort of Korean women. Specifically, we assessed if the genotypes or haplotypes of the 1451C>T, 1612G>A, and 1725G>A SNPs influence RPL risk. From this investigation, we found that all three SNPs are associated with an altered incidence of RPL. Furthermore, our haplotype analysis revealed significant differences in the *VEGF* 1451/1612/1725, *VEGF* 1451/1612, *VEGF* 1451/1725, and *VEGF* 1612/1725 haplotypes between the control subjects and patients with RPL.

In a previous study, *VEGF* 3′-UTR polymorphisms were reported to be associated with colorectal cancer. Additionally, the 1451C>T SNP was significantly associated with rectal cancer risk, and 1725G>A was correlated with metabolic syndrome risk [14]. However, *VEGF* 1451C>T was not associated with the occurrence of RPL in our study. The combined *VEGF* 3′-UTR genotypes of -1612G>A and 1725G>A were significantly increased in RPL, indicating that the effect of *VEGF* polymorphisms could partially explain RPL occurrence. Until now, no articles on 3′-UTR polymorphisms of *VEGF* and RPL have been published.

Although associations between *VEGF* polymorphisms and RPL have been reported in many studies, no studies have specifically analyzed SNPs in the *VEGF* 3′-UTR. Thus, to our knowledge, this study is the first to provide evidence that SNPs in the 3′-UTR of *VEGF* correlate with RPL.

For the subjects in our study who experienced RPL, all pregnancy loss occurred before 20 weeks gestational age. We considered that chromosomal status of the spontaneously aborted fetus could be the reason for pregnancy loss; however, this was shown not to be the case. Furthermore, our previous studies have shown no relation between SNPs and chromosomal status [15,16]. These studies excluded patients with RPL caused by thrombotic, chromosomal, hormonal, autoimmune, or anatomic factors. The selected group in this study included women with two consecutive abortions, and the type of pregnancy loss was not described. Taken together, the data in this study shows that the AA genotype of the rs1570360 polymorphism and TT genotype of the rs3025039 polymorphism can be important risk factors for RPL. The mean age of the RPL patients ranged from 27.6 to 33years (mean age 33.24 ± 4.59 years), and the control group was included in the 27.3 to 37 years age group (mean age 33.24 ± 4.59 years) [17].

We also investigated the association between other clinical variables and the *VEGF* 3′-UTR SNPs. In RPL patients, *VEGF* 1725G>A polymorphisms, specifically the G allele, are associated with higher total cholesterol counts (187.80 ± 49.42 mg/dL; *p* = 0.036). Altered hormone levels, inflammation, and problems with blood vessel formation during pregnancy may all contribute to pregnancy loss [18,19]. We therefore also measured the levels of key hormones and assessed if any of these were associated with pregnancy loss in patients with specific *VEGF* 3′-UTR polymorphisms. We found that *VEGF* 1725G>A polymorphisms, particularly the G allele, was associated with higher FBS levels (95.19 ± 17.15 mg/dL; *p* = 0.017). Interestingly, LH has been associated with *VEGF* in various in vitro studies, which found that *VEGF* synthesis increases alongside LH expression in granulosa cells [20]. For the GG allele of *VEGF* 1725G>A, FSH and LH levels were significantly different between the RPL group and the control group (Tables S1 and S2).

Hct has been shown to inhibit angiogenesis both in vitro and in vivo [21], and it is, therefore, not surprising to find a relationship between specific *VEGF* genotypes and Hct. *VEGF* and its receptors play essential roles in fetal and placental angiogenic development [22].

It has not been determined how polymorphisms in the 3′-UTR region of *VEGF* might contribute to RPL; however, we propose that they may affect microRNA (miRNA)-binding sites. One research group has previously shown altered miR-561 binding activity in response to *TS* 1494ins/del polymorphisms, which contributes to breast cancer risk. Another paper identified miRNAs that control the expression of angiogenic factors and found a number of these that regulate *VEGF* levels. Importantly, it was shown that *VEGF* levels can be modulated by these miRNAs, depending on the number of SNP combinations in its 3′-UTR [23]. Our previous case-control study also showed an association between the *VEGF* 3′-UTR SNP, +936C>T, and inferior outcome at 90 days following ischemic stroke. Additionally, we observed that the +1451C>T variant affects binding of the *VEGF* 3′-UTR by several miRNAs, which could influence *VEGF* expression [24].

In Figure S1, we show a summary of the known miRNA target sites in the 3′-UTR of the human *VEGF* mRNA sequence and indicate which of these overlap with the polymorphic loci investigated in this study. We also genotyped the *VEGF* 3′-UTR by TaqMan-assay (Table S3). Recently, several databases of predicted miRNA targets have been established, such as miRNA SNP [25], which, in combination with our genotyping data, may aid in the identification of new functional polymorphisms in the *VEGF* 3′-UTR.

A previous study demonstrated that immune function was decreased in cancer patients. *VEGF* is also a known key factor in the immune system [26]. In Figure S2, we show linkage disequilibrium patterns of *VEGF* SNPs. Regarding genetic analysis, we examined the LD pattern of three *VEGF* 3′-UTR polymorphisms (Figure S2) and found strong LD between 1451C>T (3025040) and 1612G>A (rs10434, D′ = 0.872, LOD = 6.35, R^2^ = 0.032), between 1451C>T (3025040) and 1725G>A (rs3025053, D′ = 1.000, LOD = 4.25, R^2^ = 0.014), and between 1612G>A (rs410434) and 1725G>A (rs3025053, D′ = 0.887, LOD = 32.93, R^2^ = 0.251) among the RPL subjects. *VEGF* 3′-UTR was associated with a significant increase in RPL occurrence.

Analysis of associations between environmental factors and *VEGF* genotypes in RPL patients showed statistically significant results for FSH and LH levels. Associations of FSH, *VEGF*-A, and 2-methoxyestradiol with follicular angiogenesis, growth, and atresia in mouse ovaries have previously been reported [27]. Our data show the RPL incidence according to interactions with FSH (Figure S3). We suggest that FSH may be a risk factor, associated with an increased risk of RPL in *VEGF* 1451C>T polymorphisms.

We also identified BUN, Cr, and Hct as RPL risk factors (Tables S4 and S5). Previous studies have shown that *VEGF* expression is reduced in pre-eclamptic women and in a homocysteine-treated mouse model of pre-eclampsia [28]. Based on our current findings, it is expected that there will be a correlation between *VEGF* 1612 G>A and *VEGF* expression. In addition, homocysteine, total cholesterol, PLT, BUN, and aPTT were significant risk factors of RPL (Tables S6 and S7). The balance between homocysteine and folate is an important factor in pregnancy. Homocysteine causes defects in both the neural tube and heart in embryos and increases the risk of growth abnormalities and retardation of somite development in mouse and rat embryos [29]. During pregnancy, homocysteine is a sulfur amino acid and a byproduct of the methionine bio-synthesis pathway [30].

Thus far, the association between 3′-UTR polymorphisms of *VEGF*s including *VEGF*-A and RPL has been not investigated. In previous studies, *MTHFR* 3′-untranslated region polymorphisms were shown to contribute to recurrent pregnancy loss risk [31,32,33,34].

This studies that RPL was associated with higher frequencies of the 4869G and 5488T *MTHFR* alleles. These alleles were, in turn, associated with differences in Hcy and folate levels between controls and women with RPL. Moreover, the *MTHFR* 4869G allele was associated with lower percentages of CD56+ NK cells, which has been linked with a favorable pregnancy result in women with RPL [35]. For these reasons, we conducted a study to examine the SNP genotypes and haplotypes of the 3´-UTR region, which is the microRNA (miRNA) binding site. Previous study examined four polymorphisms of the 3´-UTR of *MTHFR* in association with RPL in Korean women.

Further studies of the effects of the 3´-UTR polymorphisms are needed to determine their effect on miRNA binding and *VEGF*-A expression.

In previous study, the most important reason for the 3′-UTR is also the affected of miRNAs binding to *VEGF* 1451. The SNP of the 3′-UTR associated with *VEGF* show that *VEGF* +936C>T and +1451C>T were located in the 3ˈ-UTR of the *VEGF*-A gene, this studies hypothesized that these genetic variants may interrupt miRNA (miR-199a and miR-199b)–mRNA interactions and affect *VEGF* expression [36]. In addition, the +1451 T allele may change the conformation of the secondary structure of *VEGF*-A and may increase the binding affinities between the *VEGF*-A mRNA and the miRNAs compared with the +1451 C allele.

During pregnancy, *VEGF* is essential for the proliferation of trophoblasts, the development of embryonic vasculature and the growth of maternal and fetal blood cells in utero. Also, genetic alteration as *VEGF*-A 3′-UTR gene polymorphism has a statistical significant correlation with the severity of pre-eclampsia [24]. Therefore, we expected that polymorphisms of 3′-UTR may affect function of *VEGF*-A. We believe that our study is currently limited by the interpretation of statistical analysis of association between *VEGF* and RPL patients; however, such an association needs to be considered and should be studied further.

Based on the above data, we suggest that the *VEGF* 1451C>T, 1612G>A, and 1725G>A SNPs contribute to RPL risk. Although we identified significant genetic associations, our study had several limitations, including the following: (1) insufficient clinical information from control subjects; (2) no assessment of vascular risk factors; and (3) a control group of relatively small sample size. Therefore, in future studies, this analysis should be expanded to include a more diverse patient.

## 4. Materials and Methods

### 4.1. Subjects

The study enrolled 378 idiopathic RPL patients (mean age ± standard deviation [SD], 33.24 ± 4.59 years; body mass index [BMI], 21.49 ± 3.87) and 236 control subjects (mean age ± SD, 33.37 ± 5.81 years; BMI, 21.69 ± 3.37) between March 1999 and February 2012.

Blood samples were collected from 614 study participants including 378 patients with RPL (mean age ± standard deviation [SD], 33.24 ± 4.59 years) and 236 control subjects (mean age ± SD, 33.37 ± 5.81 years). All control patients were fertile 46, XX females who had successfully carried one or more naturally conceived pregnancies to term and who had no history of miscarriage.

All sampling occurred during the enrollment period, and written informed consent was obtained from all study participants. All patients had suffered a minimum of two consecutive spontaneous miscarriages and were diagnosed with RPL based on human chorionic gonadotropin (hCG) levels prior to 20 weeks gestation. All control patients were fertile 46, XX females that had successfully carried one or more naturally conceived pregnancies to term and that had no history of miscarriage. Exclusion criteria were RPL resulting from thrombotic, chromosomal, hormonal, autoimmune, or anatomic factors, and history of alcohol use or smoking. The CHA Bundang Medical Center Institutional Review Board (IRB-number: 2010-01-123) the study.

### 4.2. Genotyping

Blood samples were obtained on days 3–7 of the menstrual cycle at the Department of Obstetrics and Gynecology and the Fertility Center of CHA Bundang Medical Center (Seongnam, South Korea). Leukocyte DNA extraction was performed using a G-DEX II Kit (QIAGEN GmbH, Hilden, Germany) as per the manufacturer’s instructions. Real-time PCR (RG-6000, Corbett Research, Sydney, Australia) with TaqMan allelic discrimination was used to genotype the *VEGF* 1451C>T, 1612G>A, and 1725G>A SNPs. Primer Express Software (v2.0) was used to design primers and TaqMan probes, which were then purchased from Applied Biosystems (Foster City, CA, USA). The primers and probes were as follows: *VEGF* 1451C>T: 5′-ACG GAC AGA AAG ACA GAT CAC AG-3′ (forward), 5′-CCC AAA GCA CAG CAA TGT C-3′ (reverse), 5′-FAM- TGA GGA CAC CGG CTC TGA CC -TAMRA-3′ (C allele specific probe), and 5′-JOE- TGA GGA CAC TGG CTC TGA CC -TAMRA-3′ (T allele specific probe); *VEGF* 1612G>A: 5′-TTC GCT TAC TCT CAC CTG CTT C-3′ (forward), 5′-GCT GTC ATG GGC TGC TTC T-3′(reverse), 5′-FAM- CCC AGG CCA CTG GCA -TAMRA-3′ (G allele specific probe), and 5′-JOE-CCC AGG AGA CCA CTG GCA-TAMRA-3′ (A allele specific probe); *VEGF* 1725G>A: 5′-CAT GAC AGC TCC CCT TCC T-3′ (forward), 5′-TGG TTT CAA TGG TGT GAG GAC-3′(reverse), 5′-FAM- CTT CCT GGG GTG CAG CCT AA -TAMRA-3′ (G allele specific probe), and 5′-JOE-CTT CCT GGG ATG CAG CCT AA-TAMRA-3′ (A allele specific probe).

To validate the TaqMan results, DNA sequencing (ABI 3730xl, Applied Biosystems) was performed on a random 30% of the PCRs for each SNP. Quality control samples had a concordance rate of 100%. Additionally, analyses using leukocyte DNA purified by a different method (QIAamp DNA Blood Isolation Kit QIAGEN, QIAGEN GmbH, Hilden, GERMANY) yielded comparable results [37].

### 4.3. Measurement of Blood Coagulation and Folic Acid, Uric Acid, Total Cholesterol, and Homocysteine Concentrations

Samples of blood collected from RPL patients after fasting for 12 h were used for the following analyses. Blood coagulation was assessed using prothrombin time (11.59 ± 0.86 sec) (ACL TOP; Mitsubishi Chemical Medience, Tokyo, Japan), activated partial thromboplastin time (32.32 ± 4.31 sec) (ACL TOP), and platelet counts (255.43 ± 59.22 103 cells/μL) (Sysmex XE2100, Sysmex, Kobe, Japan). Folic acid levels (14.27 ± 12.00 ng/mL) were measured by competitive immunoassay (ACS: 180, Bayer Diagnostics, Tarrytown, NY, USA). Commercially available colorimetric enzymatic tests (Roche Diagnostics, Mannheim, Germany) were used to measure uric acid levels (3.77 ± 0.80 mg/dL) and total blood cholesterol (187.80 ± 49.42 mg/dL). Homocysteine concentrations (6.94 ± 2.05 μM) were measured by fluorescence polarization immunoassay (Abbott IMx, Abbott Laboratories, Abbott Park, IL, USA).

### 4.4. Peripheral Blood Mononuclear Cell (PBMC) Isolation and Peripheral Natural Killer (NK) Cell Abundances

Cell preparation tubes containing sodium citrate (Becton-Dickinson, Franklin Lakes, NJ, USA) were used to isolate PBMCs from whole blood. For storage, liquid nitrogen was used to freeze PBMCs in 10% RPMI 1640 medium (Life Technologies, Carlsbad, CA, USA), 10% dimethyl sulfoxide (Sigma-Aldrich, St. Louis, MO, USA), and 80% fetal bovine serum (FBS) (Lonza, Cologne, Germany). PBMCs were cultured in RPMI 1640 medium containing 2 mM glutamine (Life Technologies), 50 μM 2-mercaptoethanol (Sigma-Aldrich), 50 mg/mL gentamicin sulfate (Lonza), and 10% FBS. After two washes in phosphate buffered saline (Welgene, Seoul, Korea), the PBMCs were resuspended at 1 × 10^6^ cells/mL in RPMI 1640 medium supplemented with 1% sodium pyruvate (Life Technologies), 1% MEM Nonessential Amino Acids Solution (Life Technologies), and 10% FBS, followed by incubation overnight. Assays for NK cells were always conducted following incubation for 16–20 h. To determine NK cell counts, peridinin chlorophyll protein-conjugated anti-CD3, and phycoerythrin-conjugated anti-CD56 and monoclonal antibodies (BD Biosciences, San Jose, CA, USA) were added to 200 mL of diluted blood that had been incubated on ice for 20 min, and the cells were then analyzed by flow cytometry (BD FACSCalibur, BD Biosciences). The NK cell abundance was calculated as (NK cells/mL sample) = [(CD3/CD56 cell count)/ bead count] × 100.

### 4.5. Hormone Assay

Levels of luteinizing hormone (LH), follicle-stimulating hormone (FSH), prolactin, and E2 were measured in serum prepared from venipuncture blood samples collected on menstrual cycle days 2 or 3, as previously described. LH and FSH levels were measured by enzyme immunoassays (Siemens, Munich, Germany). Prolactin and E2 levels were measured by radioimmunoassays (Beckman Coulter, Brea, CA, USA). All analyses were conducted as per the manufacturers’ instructions.

### 4.6. Statistical Analysis

The associations between RPL incidence and the VEGF SNPs were evaluated by odds ratios (ORs), adjusted odds ratios (AORs), and 95% confidence intervals (95% CIs) from logistic regression and Fisher’s exact test, with adjustment for the age of the participants, as calculated by GraphPad Prism 4.0 (GraphPad Software Inc., San Diego, CA, USA) and MedCalc v12.7.1.0 (MedCalc Software, Mariakerke, Belgium). The expectation-maximization algorithm with SNPAlyze v5.1 (DYNACOM Co, Ltd., Yokohama, Japan) was used to estimate multilocus haplotype frequencies. The results for haplotypes with frequencies <1% were not shown due to lack of statistical significance.

## 5. Conclusions

In conclusion, we investigated the association between three SNPs in the 3′-UTR of the *VEGF* gene, namely 1451C>T, 1612G>A, and 1725G>A, and the prevalence of RPL in Korean women. Overall, the data reveal that these SNPs are associated with RPL susceptibility and may interact with environmental and clinical risk factors to influence the risk for developing this condition. The frequencies of 1612G>A polymorphisms significantly differed between RPL patients and controls. We also found that the *VEGF* 1612A allele decreased RPL risk by 0.654-fold. Similarly, the frequencies of 1725G>A polymorphisms in RPL patients differed from those in controls and the *VEGF* 1725A allele reduced RPL risk by 0.446-fold. Based on these results, we propose that the *VEGF* 3′-UTR 1612G>A, and 1725G>A polymorphisms are possible predisposing factors for RPL. Consequently, variants in the *VEGF* 3′-UTR may provide the first biomarkers for RPL prevention. However, future studies incorporating ethnically diverse groups of patients will be critical to confirm the validity of these results.

## Figures and Tables

**Table 1:** Clinical characteristics of RPL patients and controls.

Characteristic	Controls (*n* = 236)	RPL Patients (*n* = 378)	*p*
Age (years)	33.37 ± 5.81	33.24 ± 4.59	0.756
BMI (kg/m²)	21.69 ± 3.37	21.49 ± 3.87	0.642
Previous pregnancy losses (N)	None	3.29 ± 1.85	
RPL < 14 wk (%)	None	98.90%	
Live births (N)	1.72 ± 0.72	None	
Mean gestational age (weeks)	39.26 ± 1.66	7.38 ± 1.92	<0.0001
PLT (10^3^/μL)	237.25 ± 66.19	255.43 ± 59.22	0.007
Hct (%)	35.63 ± 4.31	37.33 ± 3.37	<0.0001
LH (mIU/mL)	3.32 ± 1.74	6.33 ± 12.21	0.011
E2 (pg/mL)	26.00 ± 14.75	35.78 ± 29.64	0.002
Homocysteine (μmol/L)	NA	6.94 ± 2.05	
Folate (ng/mL)	NA	14.27 ± 12.00	
Total cholesterol (mg/dL)	NA	187.80 ± 49.42	
Uric acid (mg/dL)	NA	3.77 ± 0.80	
CD56+ NK cells (%)	NA	18.10 ± 7.90	
PAI-1 (ng/mL)	NA	10.37 ± 5.70	
PT (sec)	11.51 ± 3.13	11.59 ± 0.86	0.727
aPTT (sec)	33.41 ± 3.74	32.32 ± 4.31	0.069
BUN (mg/dL)	NA	9.95 ± 2.79	
Creatinine (mg/dL)	NA	0.72 ± 0.12	
FBS (mg/dL)	NA	95.19 ± 17.15	
HDL (mg/dL)	NA	61.82 ± 17.63	
TG (mg/dL)	NA	175.79 ± 150.23	
FSH (mIU/mL)	8.12 ± 2.85	7.53 ± 10.63	0.566
Prolactin (ng/mL)	NA	15.57 ± 13.01	

Abbreviations: RPL, recurrent pregnancy loss; BMI, body mass index; NA, not applicable; PAI-1, plasminogen activator inhibitor-1; PLT, platelet; PT, prothrombin time; aPTT, activated partial thromboplastin time; BUN, blood urea nitrogen; FSH, follicle-stimulating hormone; E2, prostaglandin E2; LH, luteinizing hormone; Hct, hematocrit; Hcy, homocysteine; TG, triglyceride; HDL, high-density lipoprotein; FBS, fasting blood glucose.

**Table 2:** Genotype frequencies of *VEGF* gene polymorphisms between controls and RPL patients.

Genotype	Controls (*n* = 236)	RPL Patients (*n* = 378)	AOR (95% CI)	*p* ^a^	*p* ^b^
*VEGF* 1451C>T					
CC	166 (70.3)	242 (64.0)	1.000 (reference)		
CT	61 (25.8)	126 (33.3)	1.406 (0.977–2.024)	0.067	0.067
TT	9 (3.8)	10 (2.7)	0.783 (0.310–1.977)	0.605	0.908
Dominant (CC vs. CT+TT)			1.324 (0.933–1.879)	0.116	0.116
Recessive (CC+CT vs. TT)			0.693 (0.277–1.734)	0.433	0.650
C allele	393 (83.3)	610 (80.7)	1.000 (reference)		
T allele	79 (16.7)	146 (19.3)	1.187 (0.877–1.605)	0.267	0.267
HWE *P*	0.265	0.176			
*VEGF* 1612G > A					
GG	156 (66.1)	285 (75.4)	1.000 (reference)		
GA	68 (28.8)	82 (21.7)	0.652 (0.447–0.951)	0.026	0.039
AA	12 (5.1)	11 (2.9)	0.506 (0.218–1.174)	0.113	0.338
Dominant (GG vs. GA+AA)			0.630 (0.441–0.901)	0.011	0.017
Recessive (GG+GA vs. AA)			0.564 (0.244–1.300)	0.179	0.536
G allele	380 (80.5)	652 (86.2)	1.000 (reference)		
A allele	92 (19.5)	104 (13.8)	0.654 (0.481–0.891)	0.007	0.011
HWE *P*	0.208	0.095			
*VEGF* 1725G>A					
GG	199 (84.3)	348 (92.1)	1.000 (reference)		
GA	34 (14.4)	30 (7.9)	0.503 (0.299–0.848)	0.010	0.029
AA	3 (1.3)	0 (0.0)	N/A	0.995	0.995
Dominant (GG vs. GA+AA)			0.462 (0.277–0.772)	0.003	0.010
Recessive (GG+GA vs. AA)			N/A	0.993	0.993
G allele	432 (91.5)	726 (96.0)	1.000 (reference)		
A allele	40 (8.5)	30 (4.0)	0.446 (0.273–0.726)	0.001	0.004
HWE *P*	0.273	0.422			

Abbreviations: RPL, recurrent pregnancy loss; HWE, Hardy–Weinberg Equilibrium. ^a^ Adjusted by age of female participants. ^b^ False discovery rate-adjusted *p*-value for multiple hypotheses testing using the Benjamini–Hochberg method. Acceptance of statistical significance at *p* < 0.05 and 95% CI not including 1.

**Table 3:** Haplotype analysis for the *VEGF* polymorphisms 1451C>T, 1612G>A and 1725G>A in RPL patients and controls.

Haplotype	Controls (2*n* = 472)	RPL Patients (2*n* = 756)	OR (95% CI)	*p* ^a^	*p* ^b^
*VEGF* 1451/1612/1725					
C-G-G	300 (63.6)	505 (66.8)	1.000 (reference)		
C-G-A	6 (1.3)	1 (0.1)	0.099 (0.012–0.827)	0.013	0.026
C-A-G	53 (11.2)	75 (9.9)	0.841 (0.575–1.229)	0.379	0.386
C-A-A	34 (7.2)	29 (3.8)	0.507 (0.303–0.849)	0.011	0.026
T-G-G	75 (15.9)	146 (19.3)	1.156 (0.846–1.582)	0.386	0.386
T-G-A	0 (0.0)	0 (0.0)	N/A	N/A	N/A
T-A-G	4 (0.8)	0 (0.0)	0.066 (0.004–1.232)	0.020	0.033
T-A-A	0 (0.0)	0 (0.0)	N/A	N/A	N/A
*VEGF* 1451/1612					
C-G	305 (64.6)	506 (66.9)	1.000 (reference)		
C-A	88 (18.6)	104 (13.8)	0.712 (0.519–0.979)	0.040	0.060
T-G	75 (15.9)	146 (19.3)	1.173 (0.858–1.604)	0.345	0.345
T-A	4 (0.8)	0 (0.0)	0.067 (0.004–1.250)	0.020	0.060
*VEGF* 1451/1725					
C-G	353 (74.8)	580 (76.7)	1.000 (reference)		
C-A	40 (8.5)	30 (4.0)	0.457 (0.279–0.746)	0.002	0.004
T-G	79 (16.7)	146 (19.3)	1.125 (0.830–1.525)	0.490	0.490
T-A	0 (0.0)	0 (0.0)			
*VEGF* 1612/1725					
G-G	374 (79.2)	651 (86.1)	1.000 (reference)		
G-A	6 (1.3)	1 (0.1)	0.096 (0.011–0.799)	0.012	0.018
A-G	58 (12.3)	75 (9.9)	0.743 (0.525–1.071)	0.127	0.127
A-A	34 (7.2)	29 (3.8)	0.490 (0.294– 0.817)	0.007	0.018

Abbreviations: RPL, recurrent pregnancy loss; OR, odds ratio. ^a^ Fisher’s exact test. ^b^ False discovery rate-adjusted *p*-value for multiple hypotheses testing using the Benjamini–Hochberg method. Acceptance of statistical significance at *p* < 0.05 and 95% confidence interval not including 1.

**Table 4:** Combined genotype analysis for the *VEGF* polymorphisms 1451C>T, 1612G>A, and 1725G>A in RPL patients and controls.

Combined Genotype	Controls (*n* = 236)	RPL Patients (*n* = 388)	AOR (95% CI) ^b^	*p* ^a^	*p* ^b^
***VEGF* 1451C>T/*VEGF* 1612G>A**					
CC/GG	100 (42.6)	178 (45.9)	1.000 (reference)		
CC/GA	54 (22.8)	62 (16.0)	0.679 (0.437–1.055)	0.085	0.333
CC/AA	12 (5.1)	11 (2.8)	0.550 (0.233–1.295)	0.171	0.333
CT/GG	49 (20.7)	106 (27.3)	1.279 (0.841–1.946)	0.250	0.333
CT/GA	12 (5.1)	21 (5.4)	0.937 (0.436–2.011)	0.867	0.867
CT/AA	0 (0.0)	0 (0.0)	N/A	N/A	N/A
TT/GG	7 (3.0)	10 (2.6)	0.866 (0.318–2.358)	0.779	0.867
TT/GA	2 (0.8)	0 (0.0)	N/A	N/A	N/A
TT/AA	0 (0.0)	0 (0.0)	N/A	N/A	N/A
***VEGF* 1451C>T/*VEGF* 1725G>A**					
CC/GG	133 (56.5)	221 (57.0)	1.000 (reference)		
CC/GA	30 (12.7)	30 (7.7)	0.510 (0.290–0.898)	0.020	0.020
CC/AA	3 (1.3)	0 (0.0)	N/A	N/A	N/A
CT/GG	58 (24.5)	123 (31.7)	1.275 (0.871–1.865)	0.211	0.317
CT/GA	3 (1.3)	4 (1.0)	0.802 (0.176–3.652)	0.776	0.776
CT/AA	0 (0.0)	0 (0.0)	N/A	N/A	N/A
TT/GG	9 (3.8)	10 (2.6)	0.698 (0.276–1.771)	0.450	0.600
TT/GA	0 (0.0)	0 (0.0)	N/A	N/A	N/A
TT/AA	0 (0.0)	0 (0.0)	N/A	N/A	N/A
***VEGF* 1612G>A/*VEGF* 1725G>A**					
GG/GG	153 (65.0)	289 (74.5)	1.000 (reference)		
GG/GA	3 (1.3)	5 (1.3)	0.178 (0.018–1.729)	0.137	0.137
GG/AA	0 (0.0)	0 (0.0)	N/A	N/A	N/A
GA/GG	42 (17.7)	61 (15.7)	0.776 (0.497–1.210)	0.263	0.263
GA/GA	24 (10.1)	22 (5.7)	0.472 (0.258–0.866)	0.015	0.045
GA/AA	2 (0.8)	0 (0.0)	N/A	N/A	N/A
AA/GG	5 (2.1)	4 (1.0)	0.440 (0.116–1.670)	0.228	0.409
AA/GA	6 (2.5)	7 (1.8)	0.627 (0.207–1.898)	0.409	0.409
AA/AA	1 (0.4)	0 (0.0)	N/A	N/A	N/A

Abbreviations: RPL, recurrent pregnancy loss; AOR, adjusted odds ratio. ^a^ Fisher’s exact test. ^b^ False discovery rate-adjusted *p*-value for multiple hypotheses testing using the Benjamini–Hochberg method. Acceptance of statistical significance at *p* < 0.05 and 95% confidence interval not including 1. ORs and 95% confidence intervals of each specific genotype were calculated with reference to frequencies of all others.

**Table 5:** RPL incidence by interactions with environmental factor such as advanced age, BMI, PLT, PT, aPTT, Hct, FSH.

Characteristics	*VEGF* 1451C>T CC	*VEGF* 1451C>T CT+TT
Age		
<33	1.000 (reference)	1.162 (7.710–1.902)
≥33	0.880 (0.592–1.307)	1.322 (0.798–2.190)
BMI		
<25 kg/m^2^	1.000 (reference)	1.058 (0.572–1.955)
≥25 kg/m^2^	0.818 (0.469–1.426)	1.057 (0.485–2.303)
PLT		
<304.00 10^3^/μL	1.000 (reference)	1.873 (1.084–3.236)
≥304.00 10^3^/μL	2.448 (1.462–4.101)	1.408 (0.730–2.715)
PT		
>10.4 sec	1.000 (reference)	0.633 (0.314–1.277)
≤10.4 sec	4.781 (1.847–12.373)	4.159 (1.500–11.529)
aPTT		
>28.00 sec	1.000 (reference)	0.738 (0.332–1.639)
≤28.00 sec	4.195 (0.944–18.646)	1.349 (0.406–4.052)
Hct		
<37.33 %	1.000 (reference)	1.518 (0.867–2.661)
≥37.33 %	1.928 (1.166–3.188)	1.728 (0.880–3.391)
FSH		
>7.53 mIU/mL	1.000 (reference)	1.283 (0.571–2.886)
≤7.53 mIU/mL	2.238 (1.227–4.081)	4.635 (2.093–10.263)

Abbreviations: RPL, recurrent pregnancy loss; BMI, body mass index; PLT, platelet; PT, prothrombin time; aPTT, activated partial thromboplastin time; FSH, follicle-stimulating hormone; Hct, hematocrit;PT 10.04 sec, and aPTT 28.00 were lower 15% cut-off each level in RPL patients and controls. Platelet 304.00 10^3^/μL was upper 15% cut-off each level in RPL patients and controls.

**Table 6:** Clinical variables in RPL patients, stratified by *VEGF* polymorphisms 1451C>T, 1612G>A, and 1725G>A status.

Genotype	BUN (mg/dL)		Cr (mg/dL)		Total Cholesterol (mg/dL)		FBS (mg/dL)		Hct (%)		HDL (mg/dL)		LH (mIU/mL)		PT (sec)	
	Mean ± SD	*p* ^a^	Mean ± SD	*p* ^a^	Mean ± SD	*p* ^a^	Mean ± SD	*p* ^a^	Mean ± SD	*p* ^a^	Mean ± SD	*p* ^a^	Mean ± SD	*p* ^a^	Mean ± SD	*p* ^a^
*VEGF* 1451 C>T																
CC	10.09 ± 2.83	0.412	0.72 ± 0.13	0.783	189.17 ± 48.91	0.874	95.84 ± 18.63	0.691	36.88 ± 3.89	0.032	53.72 ± 13.62	0.048	5.28 ± 10.97	0.880	11.52 ± 1.90	0.636
CT	9.60 ± 2.68		0.73 ± 0.12		185.58 ± 51.66		94.25 ± 14.26		35.74 ± 3.92		69.92 ± 18.10		5.37 ± 7.67		11.63 ± 1.25	
TT	10.9 ± 3.20		0.73 ± 0.10		181.25 ± 38.65		90.00 ± 10.98		36.63 ± 4.29		N/A		3.70 ± 2.13		12.08 ± 1.68	
C allele	9.93 ± 2.78	0.492	0.72 ± 0.12	0.933	187.96 ± 49.73	0.789	95.30 ± 17.26	0.542	36.51 ± 3.93	0.924	61.82 ± 17.63	N/A	5.31 ± 10.10	0.616	11.56 ± 1.70	0.423
T allele	10.9 ± 3.20		0.73 ± 0.10		181.25 ± 38.65		90.00 ± 10.98		36.63 ± 4.29		N/A		3.70 ± 2.13		12.08 ± 1.68	
*VEGF* 1612 G>A																
GG	9.83 ± 2.74	0.647	0.72 ± 0.12	0.957	192.37 ± 50.64	0.083	96.20 ± 18.81	0.431	36.53 ± 3.96	0.993	62.65 ± 16.58	0.758	4.91 ± 6.26	<0.001	11.56 ± 1.42	0.003
GA	10.26 ± 3.05		0.72 ± 0.12		172.81 ± 43.00		92.88 ± 11.50		36.48 ± 3.95		59.66 ± 22.10		4.43 ± 2.56		11.34 ± 0.93	
AA	10.11 ± 2.12		0.71 ± 0.12		193.88 ± 51.47		91.89 ± 14.84		36.49 ± 3.81		N/A		19.40 ± 45.87		13.18 ± 5.47	
G allele	9.94 ± 2.82	0.859	0.72 ± 0.12	0.827	187.51 ± 49.46	0.723	95.35 ± 17.27	0.555	36.52 ± 3.95	0.982	61.82 ± 17.63	0.498	4.77 ± 5.48	<0.001	11.50 ± 1.31	0.001
A allele	10.11 ± 2.12		0.71 ± 0.12		193.88 ± 51.47		91.89 ± 14.84		36.49 ± 3.81		59.66 ± 22.10		19.40 ± 45.87		13.18 ± 5.47	
*VEGF* 1725 G>A																
GG	9.81 ± 2.77	0.050	0.72 ± 0.13	0.596	189.77 ± 49.02	0.148	95.62 ± 17.85	0.314	36.51 ± 3.92	0.851	63.30 ± 16.99	0.148	4.79 ± 5.69	0.030	11.49 ± 1.34	0.035
GA	11.07 ± 2.75		0.73 ± 0.09		172.75 ± 51.15		91.62 ± 9.02		36.62 ± 4.12		36.70 ± 0.00		8.53 ± 23.69		12.43 ± 3.71	
AA	N/A		N/A		N/A		N/A		34.40 ± 0.00		N/A		N/A		11.40 ± 0.00	
G allele	9.95 ± 2.79	0.057	0.72 ± 0.12	0.034	187.80 ± 49.42	0.036	95.19 ± 17.15	0.017	36.52 ± 3.94	0.208	61.82 ± 17.63	0.245	5.26 ± 9.94	0.160	11.57 ± 1.70	0.326
A allele	11.07 ± 2.75		0.73 ± 0.09		172.75 ± 51.15		91.62 ± 9.02		36.53 ± 4.05		36.7 ± 0.00		8.53 ± 23.69		12.35 ± 3.57	

Abbreviations: RPL, recurrent pregnancy loss; PT, prothrombin time; BUN, blood urea nitrogen; Hct, hematocrit; HDL, high- density lipoprotein; LH, luteinizing hormone; Cr, creatinine; FBS, fasting blood sugar; SD, standard deviation. ^a^ Calculated using ANOVA; Calculated using the Kruskal-Wallis test.

**Table 7:** Multiple linear regression analyses of clinical variables in Korean RPL patients according to the quintiles of clinical variables.

Genotype	Homocysteine Decile (μmol/L)			BUN Decile (mg/dL)			Total Cholesterol Decile (mg/dL)			Hct Decile (%)			PLT Decile (10^3^/μL)			aPTT Decile (sec)		
	*n* = 276	Coef	*p*	*n* = 192	Coef	*p*	*n* = 173	Coef	*p*	*n* = 200	Coef	*p*	*n* = 200	Coef	*p*	*n* = 206	Coef	*p*
*VEGF* 1451C>T																		
CC	174 (63.0)	(ref)		125 (65.1)	(ref)		112 (64.7)	(ref)		128 (64.0)	(ref)		128 (64.0)	(ref)		131 (63.6)	(ref)	
CT	95 (34.4)	−0.682	0.065	63 (32.8)	−0.409	0.360	57 (32.9)	−0.476	0.316	68 (34.0)	−1.058	0.014	68 (34.0)	−1.215	0.005	71 (34.5)	0.171	0.692
TT	7 (2.5)	−0.296	0.787	4 (2.1)	0.448	0.764	4 (2.3)	−0.366	0.804	4 (2.0)	−0.539	0.708	4 (2.0)	−0.156	0.918	4 (1.9)	−1.685	0.254
Dominant (CC vs. CT+TT)		−0.656	0.067		−0.358	0.414		−0.469	0.311		−1.029	0.014		−1.156	0.006		0.072	0.865
Recessive (CC+CT vs. TT)		−0.055	0.960		0.585	0.689		−0.206	0.889		−0.176	0.903		0.265	0.856		−1.745	0.233
*VEGF* 1612G>A																		
GG	204 (73.9)	(ref)		136 (70.8)	(ref)		124 (71.7)	(ref)		144 (72.0)	(ref)		144 (72.0)	(ref)		150 (72.8)	(ref)	
GA	63 (22.8)	0.053	0.897	47 (24.5)	0.271	0.581	41 (23.7)	−1.116	0.032	48 (24.0)	−0.080	0.867	48 (24.0)	−0.049	0.919	48 (23.3)	0.955	0.044
AA	9 (3.3)	1.927	0.048	9 (4.7)	0.321	0.746	8 (4.6)	0.298	0.776	8 (4.0)	−0.851	0.415	8 (4.0)	−0.132	0.901	8 (3.9)	−0.378	0.721
Dominant (GG vs. GA+AA)		0.288	0.466		0.279	0.543		−0.885	0.070		−0.190	0.674		−0.061	0.894		0.765	0.092
Recessive (GG+GA vs. AA)		1.914	0.049		0.251	0.799		0.576	0.584		−0.831	0.421		−0.120	0.909		−0.610	0.560
*VEGF* 1725G>A																		
GG	250 (90.6)	(ref)		171 (89.1)	(ref)		153 (88.4)	(ref)		182 (91.0)	(ref)		182 (91.0)	(ref)		188 (91.3)	(ref)	
GA	26 (9.4)	0.742	0.211	21 (10.9)	1.392	0.037	20 (11.6)	−1.019	0.140	18 (9.0)	−0.220	0.756	18 (9.0)	−0.477	0.504	18 (8.7)	1.260	0.077
AA	0 (0.0)	N/A	N/A	0 (0.0)	N/A	N/A	0 (0.0)	N/A	N/A	0 (0.0)	N/A	N/A	0 (0.0)	N/A	N/A	0 (0.0)	N/A	N/A
Dominant (GG vs. GA+AA)		0.742	0.211		1.392	0.037		−1.019	0.140		−0.220	0.756		−0.477	0.504		1.260	0.077
Recessive (GG+GA vs. AA)		N/A	N/A		N/A	N/A		N/A	N/A		N/A	N/A		N/A	N/A		N/A	N/A

Abbreviations: RPL, recurrent pregnancy loss; PLT, platelet; aPTT, activated partial thromboplastin time; BUN, blood urea nitrogen; Hct, hematocrit; SD, standard deviation; Coef, regression coefficients; Ref, reference.

## Supplementary Materials

Supplementary materials can be found at https://www.mdpi.com/1422-0067/20/13/3319/s1.
