# Supplementary material for: 3′-UTR Polymorphisms in the Vascular Endothelial Growth Factor Gene (VEGF) Contribute to Susceptibility to Recurrent Pregnancy Loss (RPL)
Source: Int J Mol Sci. 2019 Jul 5;20(13):3319. doi: 10.3390/ijms20133319 (PMC6651559; doi:10.3390/ijms20133319)
Supplement: Supplementary file 1 [file ijms-20-03319-s001.zip › ijms-491329-suppl/ijms-491329-suppl-tables.docx]

| **Supplementary Table 1** | | |  |  |  | |  |  |  |
| --- | --- | --- | --- | --- | --- | --- | --- | --- | --- |
| ***VEGF* genotypes and associated serum FSH, LH levels in healthy women** | | | | | |  |  |  |  |
| **Genotypes** | **FSH** | | **LH** | | |  |  |  |  |
|  | **(mean±SD)** | ***P*^a^** | **(mean±SD)** | ***P*^a^** | |  |  |  |  |
| *VEGF 1451*  CC | 8.10±2.84 | 0.944 | 3.37±1.67 | 0.608 | |  |  |  |  |
| *VEGF 1451*  CT+TT | 8.15±2.91 |  | 3.17±1.96 |  | |  |  |  |  |
| *VEGF 1612* GG | 8.16±2.88 | 0.814 | 3.34±1.79 | 0.832 | |  |  |  |  |
| *VEGF 1612* GA+AA | 8.02±2.81 |  | 3.26±1.64 |  | |  |  |  |  |
| *VEGF 1725*  GG | 8.16±2.92 | 0.680 | 3.31±1.75 | 0.982 | |  |  |  |  |
| *VEGF 1725* GA+AA | 7.85±2.46 |  | 3.32±1.76 |  | |  |  |  |  |
| Abbreviation: FSH, follicle-stimulating hormone; LH, luteinizing hormone | | | | | |  |  |  |  |
| ^a^ Independent two-sample t-test. | |  |  |  | |  |  |  |  |

| **Supplementary Table 2** | | |  |  |  |  |  |  |
| --- | --- | --- | --- | --- | --- | --- | --- | --- |
| ***VEGF* genotypes and associated serum FSH, LH levels in RPL patients** | | | | |  |  |  |  |
| **Genotypes** | **FSH** | | **LH** | |  |  |  |  |
|  | **(mean±SD)** | ***P*^a^** | **(mean±SD)** | ***P*^a^** |  |  |  |  |
| *VEGF 1451*  CC | 7.30±8.39 | 0.698 | 6.52±13.89 | 0.770 |  |  |  |  |
| *VEGF 1451*  CT+TT | 7.92±13.82 |  | 5.98±8.40 |  |  |  |  |  |
| *VEGF 1612* GG | 7.83±12.44 | 0.535 | 5.73±7.51 | 0.292 |  |  |  |  |
| *VEGF 1612* GA+AA | 6.78±3.65 |  | 7.76±19.33 |  |  |  |  |  |
| *VEGF 1725*  GG | 7.58±11.15 | 0.824 | 5.55±6.79 | 0.011 |  |  |  |  |
| *VEGF 1725* GA+AA | 7.03±4.44 |  | 12.73±31.52 |  |  |  |  |  |
| Abbreviation: FSH, follicle-stimulating hormone; LH, luteinizing hormone | | | | |  |  |  |  |
| ^a^ Independent two-sample t-test. | |  |  |  |  |  |  |  |

| **Supplementary Table 3. Information of VEGF gene polymorphisms for primer sequences** | | | |  |  |
| --- | --- | --- | --- | --- | --- |
| Polymorphisms | RS number | Position | Primer sequence | Annealing temperature | Cycle |
| VEGF 1451C>T | rs3025040 | 3'UTR | F: ACG GAC AGA AAG ACA GAT CAC AG R: CCC AAA GCA CAG CAA TGT C C probe: 5’-FAM-TGA GGA CAC C GGC TCT GAC C-TAMRA-3’ T probe: 5’-JOE-TGA GGA CAC T GGC TCT GAC C-TAMRA-3’ | 57℃ | 50 |
| VEGF 1612G>A | rs10434 | 3'UTR | F: TTC GCT TAC TCT CAC CTG CTT C R: GCT GTC ATG GGC TGC TTC T G probe: 5’-FAM-CCC AGG AG G CCA CTG GCA-TAMRA-3’ A probe: 5’-JOE-CCC AGG AG A CCA CTG GCA-TAMRA-3’ | 65℃ | 50 |
| VEGF 1725G>A | rs3025053 | 3'UTR | F: CAT GAC AGC TCC CCT TCC T R: TGG TTT CAA TGG TGT GAG GAC G probe: 5’-FAM-CTT CCT GGG G TGC AGC CTA A-TAMRA-3’ A probe: 5’-JOE-CTT CCT GGG A TGC AGC CTA A-TAMRA-3’ | 57℃ | 50 |

Note: TaqMan assay analysis

| **Supplementary Table 4. Clinical variables in RPL patients, stratified by *VEGF* polymorphisms 1451C>T, 1612G>A and 1725G>A status.** | | | | | | | | | | | | | | | | | | |
| --- | --- | --- | --- | --- | --- | --- | --- | --- | --- | --- | --- | --- | --- | --- | --- | --- | --- | --- |
| **Characteristics** | **Homocysteine (μmol/L)** | | **Folate (mg/ml)** | | **Total cholesterol (mg/dl)** | | **Uric acid (mg/dl)** | | **CD56+ NK Cell (%)** | | **PAI-1 (ng/ml)** | | **PLT(10^3^/㎕)** | | **PT (sec)** | | **aPTT (sec)** | |
|  | Mean ± SD | *P^a^* | Mean ± SD | *P^a^* | Mean ± SD | *P^a^* | Mean ± SD | *P^a^* | Mean ± SD | *P^a^* | Mean ± SD | *P^a^* | Mean ± SD | *P^a^* | Mean ± SD | *P^a^* | Mean ± SD | *P^a^* |
| ***VEGF* 1451 C>T** |  |  |  |  |  |  |  |  |  |  |  |  |  |  |  |  |  |  |
| **CC** | 7.08±2.17 | 0.310 | 14.38±13.08 | 0.701 | 189.17±48.91 | 0.874 | 3.80±0.79 | 0.205 | 18.11±8.52 | 0.754 | 11.01±5.89 | 0.278 | 251.07±61.72 | 0.070 | 11.52±1.90 | 0.636 | 32.41±4.11 | 0.171 |
| **CT** | 6.69±1.85 |  | 13.76±8.94 |  | 185.58±51.66 |  | 3.76±0.80 |  | 18.29±7.01 |  | 9.36±5.27 |  | 235.58±61.50 |  | 11.63±1.25 |  | 33.05±4.45 |  |
| **TT** | 6.70±0.90 |  | 17.96±19.33 |  | 181.25±38.65 |  | 3.08±0.94 |  | 14.73±3.52 |  | 11.50±7.48 |  | 258.28±99.70 |  | 12.08±1.68 |  | 30.30±1.37 |  |
| **C allele** | 6.95±2.07 | 0.756 | 14.16±11.78 | 0.446 | 187.96±49.73 | 0.789 | 3.79±0.79 | 0.079 | 18.18±7.96 | 0.457 | 10.34±5.68 | 0.731 | 246.03±61.99 | 0.527 | 11.56±1.70 | 0.423 | 32.64±4.24 | 0.147 |
| **T allele** | 6.70±0.90 |  | 17.96±19.33 |  | 181.25±38.65 |  | 3.08±0.94 |  | 14.73±3.52 |  | 11.50±7.48 |  | 258.28±99.70 |  | 12.08±1.68 |  | 30.30±1.37 |  |
|  |  |  |  |  |  |  |  |  |  |  |  |  |  |  |  |  |  |  |
| ***VEGF* 1612 G>A** |  |  |  |  |  |  |  |  |  |  |  |  |  |  |  |  |  |  |
| **GG** | 6.89±1.97 | 0.361 | 14.55±12.31 | 0.858 | 192.37±50.64 | 0.083 | 3.82±0.84 | 0.503 | 17.73±7.56 | 0.766^b^ | 9.85±5.47 | 0.112 | 248.01±64.35 | 0.705 | 11.56±1.42 | **0.003** | 32.41±4.25 | 0.428 |
| **GA** | 6.98±2.27 |  | 13.57±11.64 |  | 172.81±43.00 |  | 3.65±0.68 |  | 18.83±8.23 |  | 12.38±5.90 |  | 241.86±62.05 |  | 11.34±0.93 |  | 33.14±3.91 |  |
| **AA** | 7.88±2.22 |  | 13.34±7.62 |  | 193.88±51.47 |  | 3.69±0.87 |  | 22.00±13.98 |  | 8.89±9.14 |  | 247.94±53.41 |  | 13.18±5.47 |  | 32.02±5.18 |  |
| **G allele** | 6.91±2.04 | 0.163 | 14.29±12.13 | 0.836 | 187.51±49.46 | 0.723 | 3.77±0.80 | 0.768 | 17.98±7.70 | 0.318 | 10.41±5.64 | 0.650 | 246.31±63.70 | 0.917 | 11.50±1.31 | **0.001** | 32.60±4.17 | 0.654 |
| **A allele** | 7.88±2.22 |  | 13.34±7.62 |  | 193.88±51.47 |  | 3.69±0.87 |  | 22.00±13.98 |  | 8.89±9.14 |  | 247.94±53.41 |  | 13.18±5.47 |  | 32.02±5.18 |  |
|  |  |  |  |  |  |  |  |  |  |  |  |  |  |  |  |  |  |  |
| ***VEGF* 1725 G>A** |  |  |  |  |  |  |  |  |  |  |  |  |  |  |  |  |  |  |
| **GG** | 6.88±2.03 | 0.166 | 14.34±12.30 | 0.778 | 189.77±49.02 | 0.148 | 3.78±0.82 | 0.725 | 18.03±7.67 | 0.713 | 10.21±5.65 | 0.239 | 247.84±64.81 | 0.421 | 11.49±1.34 | 0.035 | 32.41±4.18 | 0.114 |
| **GA** | 7.47±2.14 |  | 13.48±7.76 |  | 172.75±51.15 |  | 3.71±0.62 |  | 18.99±10.81 |  | 12.68±6.30 |  | 236.67±50.04 |  | 12.43±3.71 |  | 34.16±4.24 |  |
| **AA** | N/A |  | N/A |  | N/A |  | N/A |  | N/A |  | N/A |  | 203.00±0.00 |  | 11.40±0.00 |  | 35.70±0.00 |  |
| **G allele** | 6.91±2.04 | 0.217 | 14.27±12.00 | 0.568 | 187.80±49.42 | 0.036 | 3.77±0.80 | 0.802 | 18.10±7.90 | 0.336 | 10.37±5.70 | 0.145 | 246.50±63.27 | 0.977 | 11.57±1.70 | 0.326 | 32.56±4.21 | 0.606 |
| **A allele** | 7.47±2.14 |  | 13.48±7.76 |  | 172.75±51.15 |  | 3.71±0.62 |  | 18.99±10.81 |  | 12.68±6.30 |  | 235.27±49.44 |  | 12.35±3.57 |  | 34.28±4.09 |  |
| **Abbreviations: RPL, recurrent pregnancy loss; PAI-1, plasminogen activator inhibitor-1; PLT, platelet; PT, prothrombin time; aPTT, activated partial thromboplastin time; BUN, blood urea nitrogen; cr, creatine; FBS, diabetes mellitus test; Hct, hematocrit; HDL, high density lipoprotein; TG, triglycerides; FSH, follicle stimulating hormone; LH, Luteinizing hormone; SD, standard deviation. ^a^Calculated using ANOVA; ^b^Calculated using the Kruskal-Wallis test.** | | | | | | | | | | | | | | | | | | |
|  |  |  |  |  |  |  |  |  |  |  |  |  |  |  |  |  |  |  |

| **Supplementary Table 5. Clinical variables in RPL patients, stratified by *VEGF* polymorphisms 1451C>T, 1612G>A and 1725G>A status.** | | | | | | | | | | | | | | | | | | |  |  |
| --- | --- | --- | --- | --- | --- | --- | --- | --- | --- | --- | --- | --- | --- | --- | --- | --- | --- | --- | --- | --- |
| **Characteristics** | **BUN (mg/dl)** | | **cr (mg/dl)** | | **FBS (mg/dl)** | | **Hct (%)** | | **HDL (mg/dl)** | | **TG (mg/dl)** | | **FSH (mIU/mL)** | | **LH (mIU/mL)** | | **E2 (pg/mL)** | | **Prolactin (ng/mL)** | |
|  | Mean ± SD | *P^a^* | Mean ± SD | *P^a^* | Mean ± SD | *P^a^* | Mean ± SD | *P^a^* | Mean ± SD | *P^a^* | Mean ± SD | *P^a^* | Mean ± SD | *P^a^* | Mean ± SD | *P^a^* | Mean ± SD | *P^a^* | Mean ± SD | *P^a^* |
| ***VEGF* 1451 C>T** |  |  |  |  |  |  |  |  |  |  |  |  |  |  |  |  |  |  |  |  |
| **CC** | 10.09±2.83 | 0.412 | 0.72±0.13 | 0.783 | 95.84±18.63 | 0.691 | 36.88±3.89 | 0.032 | 53.72±13.62 | 0.048 | 177.47±159.35 | 0.896 | 7.63±6.74 | 0.889 | 5.28±10.97 | 0.880 | 30.64±19.40 | 0.483 | 15.43±13.56 | 0.968 |
| **CT** | 9.60±2.68 |  | 0.73±0.12 |  | 94.25±14.26 |  | 35.74±3.92 |  | 69.92±18.10 |  | 172.50±133.76 |  | 8.09±12.38 |  | 5.37±7.67 |  | 34.74±35.33 |  | 15.75±12.40 |  |
| **TT** | 10.9±3.20 |  | 0.73±0.10 |  | 90.00±10.98 |  | 36.63±4.29 |  | N/A |  | N/A |  | 7.09±2.28 |  | 3.70±2.13 |  | 31.45±26.78 |  | 16.61±8.49 |  |
| **C allele** | 9.93±2.78 | 0.492 | 0.72±0.12 | 0.933 | 95.30±17.26 | 0.542 | 36.51±3.93 | 0.924 | 61.82±17.63 | N/A | 175.79±150.23 | N/A | 7.76±8.78 | 0.810 | 5.31±10.10 | 0.616 | 31.84±25.15 | 0.961 | 15.54±13.14 | 0.843 |
| **T allele** | 10.9±3.20 |  | 0.73±0.10 |  | 90.00±10.98 |  | 36.63±4.29 |  | N/A |  | N/A |  | 7.09±2.28 |  | 3.70±2.13 |  | 31.45±26.78 |  | 16.61±8.49 |  |
|  |  |  |  |  |  |  |  |  |  |  |  |  |  |  |  |  |  |  |  |  |
| ***VEGF* 1612 G>A** |  |  |  |  |  |  |  |  |  |  |  |  |  |  |  |  |  |  |  |  |
| **GG** | 9.83±2.74 | 0.647 | 0.72±0.12 | 0.957 | 96.20±18.81 | 0.431 | 36.53±3.96 | 0.993 | 62.65±16.58 | 0.758 | 185.16±151.24 | 0.603 | 7.95±10.10 | 0.730 | 4.91±6.26 | < 0.001 | 31.13±26.63 | 0.787 | 16.81±14.54 | 0.240^b^ |
| **GA** | 10.26±3.05 |  | 0.72±0.12 |  | 92.88±11.50 |  | 36.48±3.95 |  | 59.66±22.10 |  | 140.39±152.53 |  | 7.11±2.82 |  | 4.43±2.56 |  | 33.46±21.56 |  | 12.52±7.36 |  |
| **AA** | 10.11±2.12 |  | 0.71±0.12 |  | 91.89±14.84 |  | 36.49±3.81 |  | N/A |  | 143.50±139.30 |  | 8.48±6.59 |  | 19.40±45.87 |  | 32.63±23.70 |  | 12.79±8.98 |  |
| **G allele** | 9.94±2.82 | 0.859 | 0.72±0.12 | 0.827 | 95.35±17.27 | 0.555 | 36.52±3.95 | 0.982 | 61.82±17.63 | 0.498 | 176.73±151.39 | 0.760 | 7.72±8.71 | 0.786 | 4.77±5.48 | < 0.001 | 31.80±25.25 | 0.923 | 15.65±13.12 | 0.596 |
| **A allele** | 10.11±2.12 |  | 0.71±0.12 |  | 91.89±14.84 |  | 36.49±3.81 |  | 59.66±22.10 |  | 143.50±139.30 |  | 8.48±6.59 |  | 19.40±45.87 |  | 32.63±23.70 |  | 12.79±8.98 |  |
|  |  |  |  |  |  |  |  |  |  |  |  |  |  |  |  |  |  |  |  |  |
| ***VEGF* 1725 G>A** |  |  |  |  |  |  |  |  |  |  |  |  |  |  |  |  |  |  |  |  |
| **GG** | 9.81±2.77 | 0.050 | 0.72±0.13 | 0.596 | 95.62±17.85 | 0.314 | 36.51±3.92 | 0.851 | 63.30±16.99 | 0.148 | 171.54±144.01 | 0.333 | 7.79±9.13 | 0.796 | 4.79±5.69 | 0.030 | 32.18±26.09 | 0.555 | 15.94±13.39 | 0.206 |
| **GA** | 11.07±2.75 |  | 0.73±0.09 |  | 91.62±9.02 |  | 36.62±4.12 |  | 36.70±0.00 |  | 247.00±251.51 |  | 7.40±3.67 |  | 8.53±23.69 |  | 29.51±17.92 |  | 11.97±7.82 |  |
| **AA** | N/A |  | N/A |  | N/A |  | 34.40±0.00 |  | N/A |  | N/A |  | N/A |  | N/A |  | N/A |  | N/A |  |
| **G allele** | 9.95±2.79 | 0.057 | 0.72±0.12 | 0.034 | 95.19±17.15 | 0.017 | 36.52±3.94 | 0.208 | 61.82±17.63 | 0.245 | 175.79±150.23 | 0.717 | 7.74±8.64 | 0.733 | 5.26±9.94 | 0.160 | 31.83±25.16 | 0.782 | 15.57±13.01 | 0.176 |
| **A allele** | 11.07±2.75 |  | 0.73±0.09 |  | 91.62±9.02 |  | 36.53±4.05 |  | 36.7±0.00 |  | 247.00±251.51 |  | 7.40±3.67 |  | 8.53±23.69 |  | 29.51±17.92 |  | 11.97±7.82 |  |
| **Abbreviations: RPL, recurrent pregnancy loss; PAI-1, plasminogen activator inhibitor-1; PLT, platelet; PT, prothrombin time; aPTT, activated partial thromboplastin time; BUN, blood urea nitrogen; cr, creatine; FBS, diabetes mellitus test; Hct, hematocrit; HDL, high density lipoprotein; TG, triglycerides; FSH, follicle stimulating hormone; LH, Luteinizing hormone; SD, standard deviation. ^a^Calculated using ANOVA; ^b^Calculated using the Kruskal-Wallis test.** | | | | | | | | | | | | | | | | | | |  |  |

| **Supplementary Table 6. Multiple linear regression analyses of clinical variables in Korean RPL patients according to the quintiles of clinical variables.** | | | | | | | | | | | | | | | | | | | | | | | | | | | |
| --- | --- | --- | --- | --- | --- | --- | --- | --- | --- | --- | --- | --- | --- | --- | --- | --- | --- | --- | --- | --- | --- | --- | --- | --- | --- | --- | --- |
| **Characteristics** | **Homocysteine decile^a^ (mmol/L)** | | | **Folate decile^b^ (mg/ml)** | | | **Total cholesterol decile^c^ (mg/dl)** | | | **Uric acid decile^d^ (mg/dl)** | | | **CD56+ NK Cell decile^e^ (%)** | | | **PAI-1 decile^f^ (ng/ml)** | | | **PLT decile^g^ (10^3^/㎕)** | | | **PT decile^h^ (sec)** | | | **aPTT decile^i^ (sec)** | | |
|  | n = 276 | Coef | *P* | n = 220 | Coef | *P* | n = 173 | Coef | *P* | n = 170 | Coef | *P* | n = 131 | Coef | *P* | n = 125 | Coef | *P* | n = 200 | Coef | *P* | n = 204 | Coef | *P* | n = 206 | Coef | *P* |
| ***VEGF* 1451 C>T** |  |  |  |  |  |  |  |  |  |  |  |  |  |  |  |  |  |  |  |  |  |  |  |  |  |  |  |
| **CC** | 174 (63.0) | (ref) |  | 139 (63.2) | (ref) |  | 112 (64.7) | (ref) |  | 112 (65.9) | (ref) |  | 80 (61.1) | (ref) |  | 73 (58.4) | (ref) |  | 128 (64.0) | (ref) |  | 130 (63.7) | (ref) |  | 131 (63.6) | (ref) |  |
| **CT** | 95 (34.4) | -0.682 | 0.065 | 75 (34.1) | -0.022 | 0.958 | 57 (32.9) | -0.476 | 0.316 | 54 (31.8) | -2.071 | 0.163 | 48 (36.6) | 0.400 | 0.462 | 49 (39.2) | -0.787 | 0.122 | 68 (34.0) | -1.215 | 0.005 | 70 (34.3) | 0.642 | 0.132 | 71 (34.5) | 0.171 | 0.692 |
| **TT** | 7 (2.5) | -0.296 | 0.787 | 6 (2.7) | 0.185 | 0.877 | 4 (2.3) | -0.366 | 0.804 | 4 (2.4) | -0.132 | 0.775 | 3 (2.3) | -0.892 | 0.615 | 3 (2.4) | -0.018 | 0.991 | 4 (2.0) | -0.156 | 0.918 | 4 (2.0) | -0.865 | 0.550 | 4 (1.9) | -1.685 | 0.254 |
| **Dominant (CC vs. CT+TT)** |  | -0.656 | 0.067 |  | -0.007 | 0.987 |  | -0.469 | 0.311 |  | -2.075 | 0.150 |  | 0.324 | 0.541 |  | -0.743 | 0.137 |  | -1.156 | 0.006 |  | 0.560 | 0.180 |  | 0.072 | 0.865 |
| **Recessive (CC+CT vs. TT)** |  | -0.055 | 0.960 |  | 0.193 | 0.872 |  | -0.206 | 0.889 |  | -0.698 | 0.169 |  | -1.042 | 0.547 |  | 0.298 | 0.854 |  | 0.265 | 0.856 |  | -1.090 | 0.452 |  | -1.745 | 0.233 |
|  |  |  |  |  |  |  |  |  |  |  |  |  |  |  |  |  |  |  |  |  |  |  |  |  |  |  |  |
| ***VEGF* 1612 G>A** |  |  |  |  |  |  |  |  |  |  |  |  |  |  |  |  |  |  |  |  |  |  |  |  |  |  |  |
| **GG** | 204 (73.9) | (ref) |  | 160 (72.7) | (ref) |  | 124 (71.7) | (ref) |  | 120 (70.6) | (ref) |  | 98 (74.8) | (ref) |  | 95 (76.0) | (ref) |  | 144 (72.0) | (ref) |  | 148 (72.5) | (ref) |  | 150 (72.8) | (ref) |  |
| **GA** | 63 (22.8) | 0.053 | 0.897 | 53 (24.1) | -0.334 | 0.465 | 41 (23.7) | -1.116 | 0.032 | 42 (24.7) | -0.733 | 0.488 | 29 (22.1) | 0.366 | 0.552 | 27 (21.6) | 1.043 | 0.078 | 48 (24.0) | -0.049 | 0.919 | 48 (23.5) | 0.160 | 0.736 | 48 (23.3) | 0.955 | 0.044 |
| **AA** | 9 (3.3) | 1.927 | 0.048 | 7 (3.2) | 0.011 | 0.992 | 8 (4.6) | 0.298 | 0.776 | 8 (4.7) | -0.703 | 0.142 | 4 (3.1) | 0.495 | 0.742 | 3 (2.4) | -1.179 | 0.469 | 8 (4.0) | -0.132 | 0.901 | 8 (3.9) | 0.618 | 0.553 | 8 (3.9) | -0.378 | 0.721 |
| **Dominant (GG vs. GA+AA)** |  | 0.288 | 0.466 |  | -0.294 | 0.503 |  | -0.885 | 0.070 |  | -0.553 | 0.593 |  | 0.381 | 0.522 |  | 0.821 | 0.154 |  | -0.061 | 0.894 |  | 0.225 | 0.617 |  | 0.765 | 0.092 |
| **Recessive (GG+GA vs. AA)** |  | 1.914 | 0.049 |  | 0.095 | 0.932 |  | 0.576 | 0.584 |  | -0.257 | 0.706 |  | 0.411 | 0.785 |  | -1.410 | 0.382 |  | -0.120 | 0.909 |  | 0.579 | 0.576 |  | -0.610 | 0.560 |
|  |  |  |  |  |  |  |  |  |  |  |  |  |  |  |  |  |  |  |  |  |  |  |  |  |  |  |  |
| ***VEGF* 1725 G>A** |  |  |  |  |  |  |  |  |  |  |  |  |  |  |  |  |  |  |  |  |  |  |  |  |  |  |  |
| **GG** | 250 (90.6) | (ref) |  | 203 (92.3) | (ref) |  | 153 (88.4) | (ref) |  | 150 (88.2) | (ref) |  | 121 (92.4) | (ref) |  | 117 (93.6) | (ref) |  | 182 (91.0) | (ref) |  | 186 (91.2) | (ref) |  | 188 (91.3) | (ref) |  |
| **GA** | 26 (9.4) | 0.742 | 0.211 | 17 (7.7) | 0.118 | 0.871 | 20 (11.6) | -1.019 | 0.140 | 20 (11.8) | -0.257 | 0.706 | 10 (7.6) | 0.161 | 0.869 | 8 (6.4) | 0.934 | 0.354 | 18 (9.0) | -0.477 | 0.504 | 18 (8.8) | 1.052 | 0.137 | 18 (8.7) | 1.260 | 0.077 |
| **AA** | 0 (0.0) | N/A | N/A | 0 (0.0) | N/A | N/A | 0 (0.0) | N/A | N/A | 0 (0.0) | N/A | N/A | 0 (0.0) | N/A | N/A | 0 (0.0) | N/A | N/A | 0 (0.0) | N/A | N/A | 0 (0.0) | N/A | N/A | 0 (0.0) | N/A | N/A |
| **Dominant (GG vs. GA+AA)** |  | 0.742 | 0.211 |  | 0.118 | 0.871 |  | -1.019 | 0.140 |  | -0.257 | 0.706 |  | 0.161 | 0.869 |  | 0.934 | 0.354 |  | -0.477 | 0.504 |  | 1.052 | 0.137 |  | 1.260 | 0.077 |
| **Recessive (GG+GA vs. AA)** |  | N/A | N/A |  | N/A | N/A |  | N/A | N/A |  | N/A | N/A |  | N/A | N/A |  | N/A | N/A |  | N/A | N/A |  | N/A | N/A |  | N/A | N/A |
| Note: RPL, recurrent pregnancy loss; PAI-1, plasminogen activator inhibitor-1; PLT, platelet; PT, prothrombin time; aPTT, activated partial thromboplastin time; BUN, blood urea nitrogen; cr, creatine; FBS, diabetes mellitus test; Hct, hematocrit; HDL, high density lipoprotein; TG, triglycerides; FSH, follicle stimulating hormone; LH, Luteinizing hormone; SD, standard deviation; Coef = regression coefficients; Ref = reference. aHomocysteine 10 quintiles: Hcy≤4.69, 4.69<Hcy≤5.46, 5.46<Hcy≤5.9, 5.9<Hcy≤6.31, 6.31<Hcy≤6.73, 6.73<Hcy≤7.2, 7.2<Hcy≤7.55, 7.55<Hcy≤8.2, 8.2<Hcy≤9.22, Hcy>9.22; bFolate 10 quintiles: folate≤5.34, 5.34<folate≤6.72, 6.72<folate≤7.82, 7.79<folate≤9.62, 9.62<folate≤11.24, 11.24<folate≤13.62, 13.62<folate≤16.64, 16.64<folate≤19.76, 19.76<folate≤22.88, folate>22.88; cTotal cholesterol 10 quintiles: T. chol≤137, 137<T. chol≤147, 147<T. chol≤158, 158<T. chol≤165, 165<T. chol≤179, 179<T. chol≤185, 185<T. chol≤201, 201<T. chol≤230, 230<T. chol≤261, T. chol>261; dUric acid: Uric acid≤2.8, 2.8<Uric acid≤3.1, 3.1<Uric acid≤3.4, 3.4<Uric acid≤3.5, 3.5<Uric acid≤3.7, 3.7<Uric acid≤3.9, 3.9<Uric acid≤4.1, 4.1<Uric acid≤4.4, 4.4<Uric acid≤4.8, Uric acid>4.8; eCD56+ NK Cell: NK cells≤9, 9<NK cells≤11, 11<NK cells≤13, 13<NK cells≤15, 15<NK cells≤17, 17<NK cells≤19.5, 19.5<NK cells≤21.5, 21.5<NK cells≤25, 25<NK cells≤30, NK cells>30;  fPAI-1: PAI-1≤4.24, 4.24<PAI-1≤5.74, 5.74<PAI-1≤6.65, 6.65<PAI-1≤7.5, 7.5<PAI-1≤8.67, 8.67<PAI-1≤10.19, 10.19<PAI-1≤12.79, 12.79<PAI-1≤17.805, 17.805<PAI-1≤20, PAI-1>20; gPlatelet count 10 quintiles: PLT≤186, 186<PLT≤206, 206<PLT≤222.5, 222.5<PLT≤237, 237<PLT≤250.5, 250.5<PLT≤265, 265<PLT≤277.5, 277.5<PLT≤301.5, 301.5<PLT≤324, PLT>324;  hprothrombin time 10 quintiles: PT≤10.5, 10.5<PT≤11, 11<PT≤11.2, 11.2<PT≤11.4, 11.4<PT≤11.6, 11.6<PT≤11.8, 11.8<PT≤11.9, 11.9<PT≤12.3, 12.3<PT≤12.6, PT>12.6; iactivated partial thromboplastin time: aPTT≤26.9, 26.9<aPTT≤28.6, 28.6<aPTT≤29.7, 29.7<aPTT≤30.85, 30.85<aPTT≤32.025, 32.025<aPTT≤33.2, 33.2<aPTT≤34.5, 34.5<aPTT≤36.05, 36.05<aPTT≤37.55, aPTT>37.55 jBUN: BUN≤0.6, 0.6<BUN≤0.6, 0.6<BUN≤0.7, 0.7<BUN≤0.7, 0.7<BUN≤0.7, 0.7<BUN≤0.7, 0.7<BUN≤0.8, 0.8<BUN≤0.8, 0.8<BUN≤0.9, BUN>0.9; kcr: cr≤80, 80<cr≤83, 83<cr≤87, 87<cr≤90, 90<cr≤94, 94<cr≤97, 97<cr≤98, 98<cr≤102, 102<cr≤112, cr>112; lFBS: FBS≤32.6, 32.6<FBS≤35.1, 35.1<FBS≤36.1, 36.1<FBS≤36.8, 36.8<FBS≤37.5, 37.5<FBS≤38.3, 38.3<FBS≤39.1, 39.1<FBS≤39.9, 39.9<FBS≤41.4, FBS>41.4; mHct: Hct≤37.8, 37.8<Hct≤46.9, 46.9<Hct≤52.6, 52.6<Hct≤57.3, 57.3<Hct≤59.4, 59.4<Hct≤62.1, 62.1<Hct≤66.3, 66.3<Hct≤76.1, 76.1<Hct≤87.4, Hct>87.4; nHDL: HDL≤50, 50<HDL≤60, 60<HDL≤71, 71<HDL≤90, 90<HDL≤109, 109<HDL≤138, 138<HDL≤211, 211<HDL≤279, 279<HDL≤401, HDL>401; oTG: TG≤2.62, 2.62<TG≤4.44, 4.44<TG≤5.01, 5.01<TG≤5.55, 5.55<TG≤6.04, 6.04<TG≤6.42, 6.42<TG≤7.35, 7.35<TG≤8.16, 8.16<TG≤9.98, TG>9.98; pFSH: FSH≤1.88, 1.88<FSH≤2.92, 2.92<FSH≤3.34, 3.34<FSH≤3.88, 3.88<FSH≤4.55, 4.55<FSH≤5, 5<FSH≤5.74, 5.74<FSH≤7.04, 7.04<FSH≤9.8, FSH>9.8; qLH: LH≤11, 11<LH≤16.3, 16.3<LH≤21.5, 21.5<LH≤25.7, 25.7<LH≤29.85, 29.85<LH≤36, 36<LH≤40.3, 40.3<LH≤46.5, 46.5<LH≤61.2, LH>61.2; rE2: E2≤9, 9<E2≤11, 11<E2≤13, 13<E2≤15, 15<E2≤17, 17<E2≤19.5, 19.5<E2≤21.5, 21.5<E2≤25, 25<E2≤30, E2>30; sProlactin: Prolactin≤6.42, 6.42<Prolactin≤7.44, 7.44<Prolactin≤8.72, 8.72<Prolactin≤10.22, 10.22<Prolactin≤11.83, 11.83<Prolactin≤13.9, 13.9<Prolactin≤15.66, 15.66<Prolactin≤19.36, 19.36<Prolactin≤30.91, Prolactin>30.91; | | | | | | | | | | | | | | | | | | | | | | | | | | | |

| **Supplementary Table 7. Multiple linear regression analyses of clinical variables in Korean RPL patients according to the quintiles of clinical variables.** | | | | | | | | | | | | | | | | | | | | | | | | | | | |  |  |  |
| --- | --- | --- | --- | --- | --- | --- | --- | --- | --- | --- | --- | --- | --- | --- | --- | --- | --- | --- | --- | --- | --- | --- | --- | --- | --- | --- | --- | --- | --- | --- |
| **Characteristics** | **BUN decile^j^ (mg/dl)** | | | **cr decile^k^ (mg/dl)** | | | **FBS decile^l^ (mg/dl)** | | | **Hct decile^m^ (%)** | | | **HDL decile^n^ (mg/dl)** | | | **TG decile^o^ (mg/dl)** | | | **FSH decile^p^ (mIU/mL)** | | | **LH decile^q^ (mIU/mL)** | | | **E2 decile^r^ (pg/mL)** | | | **Prolactin decile^s^ (ng/mL)** | | |
|  | n = 192 | Coef | *P* | n = 191 | Coef | *P* | n = 197 | Coef | *P* | n = 200 | Coef | *P* | n = 18 | Coef | *P* | n = 71 | Coef | *P* | n = 193 | Coef | *P* | n = 194 | Coef | *P* | n = 164 | Coef | *P* | n = 203 | Coef | *P* |
| ***VEGF* 1451 C>T** |  |  |  |  |  |  |  |  |  |  |  |  |  |  |  |  |  |  |  |  |  |  |  |  |  |  |  |  |  |  |
| **CC** | 125 (65.1) | (ref) |  | 124 (64.9) | (ref) |  | 128 (65.0) | (ref) |  | 128 (64.0) | (ref) |  | 9 (50.0) | (ref) |  | 47 (66.2) | (ref) |  | 124 (64.2) | (ref) |  | 125 (64.4) | (ref) |  | 104 (63.4) | (ref) |  | 129 (63.5) | (ref) |  |
| **CT** | 63 (32.8) | -0.409 | 0.360 | 63 (33.0) | 0.266 | 0.565 | 65 (33.0) | -0.377 | 0.387 | 68 (34.0) | -1.058 | 0.014 | 9 (50.0) | 2.111 | 0.127 | 24 (33.8) | 0.180 | 0.809 | 64 (33.2) | -0.132 | 0.768 | 64 (33.0) | -0.129 | 0.771 | 55 (33.5) | 0.021 | 0.966 | 68 (33.5) | 0.142 | 0.745 |
| **TT** | 4 (2.1) | 0.448 | 0.764 | 4 (2.1) | 0.274 | 0.859 | 4 (2.0) | -1.000 | 0.484 | 4 (2.0) | -0.539 | 0.708 | 0 (0.0) | N/A | N/A | 0 (0.0) | N/A | N/A | 5 (2.6) | 1.324 | 0.315 | 5 (2.6) | -0.120 | 0.926 | 5 (3.0) | 0.348 | 0.793 | 6 (3.0) | 1.446 | 0.224 |
| **Dominant (CC vs. CT+TT)** |  | -0.358 | 0.414 |  | 0.267 | 0.555 |  | -0.413 | 0.335 |  | -1.029 | 0.014 |  | 2.111 | 0.127 |  | 0.180 | 0.809 |  | -0.027 | 0.951 |  | -0.129 | 0.766 |  | 0.048 | 0.918 |  | 0.248 | 0.557 |
| **Recessive (CC+CT vs. TT)** |  | 0.585 | 0.689 |  | 0.185 | 0.903 |  | -0.873 | 0.547 |  | -0.176 | 0.903 |  | N/A | N/A |  | N/A | N/A |  | 1.369 | 0.295 |  | -0.076 | 0.954 |  | 0.341 | 0.795 |  | 1.397 | 0.243 |
|  |  |  |  |  |  |  |  |  |  |  |  |  |  |  |  |  |  |  |  |  |  |  |  |  |  |  |  |  |  |  |
| ***VEGF* 1612 G>A** |  |  |  |  |  |  |  |  |  |  |  |  |  |  |  |  |  |  |  |  |  |  |  |  |  |  |  |  |  |  |
| **GG** | 136 (70.8) | (ref) |  | 135 (70.7) | (ref) |  | 140 (71.1) | (ref) |  | 144 (72.0) | (ref) |  | 13 (72.2) | (ref) |  | 56 (78.9) | (ref) |  | 136 (70.5) | (ref) |  | 137 (70.6) | (ref) |  | 113 (68.9) | (ref) |  | 144 (70.9) | (ref) |  |
| **GA** | 47 (24.5) | 0.271 | 0.581 | 47 (24.6) | -0.014 | 0.978 | 48 (24.4) | -0.319 | 0.506 | 48 (24.0) | -0.080 | 0.867 | 5 (27.8) | -0.385 | 0.810 | 13 (18.3) | -1.217 | 0.177 | 50 (25.9) | 0.460 | 0.335 | 50 (25.8) | 0.565 | 0.236 | 45 (27.4) | 0.082 | 0.872 | 53 (26.1) | -0.720 | 0.121 |
| **AA** | 9 (4.7) | 0.321 | 0.746 | 9 (4.7) | -0.222 | 0.828 | 9 (4.6) | -0.687 | 0.497 | 8 (4.0) | -0.851 | 0.415 | 0 (0.0) | N/A | N/A | 2 (2.8) | -1.179 | 0.581 | 7 (3.6) | -0.360 | 0.752 | 7 (3.6) | 2.030 | 0.077 | 6 (3.7) | 1.093 | 0.367 | 6 (3.0) | -1.201 | 0.335 |
| **Dominant (GG vs. GA+AA)** |  | 0.279 | 0.543 |  | -0.048 | 0.920 |  | -0.377 | 0.403 |  | -0.190 | 0.674 |  | -0.385 | 0.810 |  | -1.212 | 0.157 |  | 0.359 | 0.431 |  | 0.745 | 0.100 |  | 0.201 | 0.681 |  | -0.769 | 0.084 |
| **Recessive (GG+GA vs. AA)** |  | 0.251 | 0.799 |  | -0.219 | 0.830 |  | -0.605 | 0.536 |  | -0.831 | 0.421 |  | N/A | N/A |  | -0.949 | 0.655 |  | -0.484 | 0.664 |  | 1.879 | 0.089 |  | 1.070 | 0.373 |  | -1.008 | 0.400 |
|  |  |  |  |  |  |  |  |  |  |  |  |  |  |  |  |  |  |  |  |  |  |  |  |  |  |  |  |  |  |  |
| ***VEGF* 1725 G>A** |  |  |  |  |  |  |  |  |  |  |  |  |  |  |  |  |  |  |  |  |  |  |  |  |  |  |  |  |  |  |
| **GG** | 171 (89.1) | (ref) |  | 170 (89.0) | (ref) |  | 176 (89.3) | (ref) |  | 182 (91.0) | (ref) |  | 17 (94.4) | (ref) |  | 67 (94.4) | (ref) |  | 172 (89.1) | (ref) |  | 173 (89.2) | (ref) |  | 145 (88.4) | (ref) |  | 184 (90.6) | (ref) |  |
| **GA** | 21 (10.9) | 1.392 | 0.037 | 21 (11.0) | 0.230 | 0.739 | 21 (10.7) | -0.398 | 0.548 | 18 (9.0) | -0.220 | 0.756 | 1 (5.6) | -4.529 | 0.134 | 4 (5.6) | 0.612 | 0.689 | 21 (10.9) | 0.065 | 0.923 | 21 (10.8) | 1.177 | 0.076 | 19 (11.6) | -0.233 | 0.741 | 19 (9.4) | -0.992 | 0.153 |
| **AA** | 0 (0.0) | N/A | N/A | 0 (0.0) | N/A | N/A | 0 (0.0) | N/A | N/A | 0 (0.0) | N/A | N/A | 0 (0.0) | N/A | N/A | 0 (0.0) | N/A | N/A | 0 (0.0) | N/A | N/A | 0 (0.0) | N/A | N/A | 0 (0.0) | N/A | N/A | 0 (0.0) | N/A | N/A |
| **Dominant (GG vs. GA+AA)** |  | 1.392 | 0.037 |  | 0.230 | 0.739 |  | -0.398 | 0.548 |  | -0.220 | 0.756 |  | -4.529 | 0.134 |  | 0.612 | 0.689 |  | 0.065 | 0.923 |  | 1.177 | 0.076 |  | -0.233 | 0.741 |  | -0.992 | 0.153 |
| **Recessive (GG+GA vs. AA)** |  | N/A | N/A |  | N/A | N/A |  | N/A | N/A |  | N/A | N/A |  | N/A | N/A |  | N/A | N/A |  | N/A | N/A |  | N/A | N/A |  | N/A | N/A |  | N/A | N/A |
| Note: RPL, recurrent pregnancy loss; PAI-1, plasminogen activator inhibitor-1; PLT, platelet; PT, prothrombin time; aPTT, activated partial thromboplastin time; BUN, blood urea nitrogen; cr, creatine; FBS, diabetes mellitus test; Hct, hematocrit; HDL, high density lipoprotein; TG, triglycerides; FSH, follicle stimulating hormone; LH, Luteinizing hormone; SD, standard deviation; Coef = regression coefficients; Ref = reference. aHomocysteine 10 quintiles: Hcy≤4.69, 4.69<Hcy≤5.46, 5.46<Hcy≤5.9, 5.9<Hcy≤6.31, 6.31<Hcy≤6.73, 6.73<Hcy≤7.2, 7.2<Hcy≤7.55, 7.55<Hcy≤8.2, 8.2<Hcy≤9.22, Hcy>9.22; bFolate 10 quintiles: folate≤5.34, 5.34<folate≤6.72, 6.72<folate≤7.82, 7.79<folate≤9.62, 9.62<folate≤11.24, 11.24<folate≤13.62, 13.62<folate≤16.64, 16.64<folate≤19.76, 19.76<folate≤22.88, folate>22.88; cTotal cholesterol 10 quintiles: T. chol≤137, 137<T. chol≤147, 147<T. chol≤158, 158<T. chol≤165, 165<T. chol≤179, 179<T. chol≤185, 185<T. chol≤201, 201<T. chol≤230, 230<T. chol≤261, T. chol>261; dUric acid: Uric acid≤2.8, 2.8<Uric acid≤3.1, 3.1<Uric acid≤3.4, 3.4<Uric acid≤3.5, 3.5<Uric acid≤3.7, 3.7<Uric acid≤3.9, 3.9<Uric acid≤4.1, 4.1<Uric acid≤4.4, 4.4<Uric acid≤4.8, Uric acid>4.8; eCD56+ NK Cell: NK cells≤9, 9<NK cells≤11, 11<NK cells≤13, 13<NK cells≤15, 15<NK cells≤17, 17<NK cells≤19.5, 19.5<NK cells≤21.5, 21.5<NK cells≤25, 25<NK cells≤30, NK cells>30;  fPAI-1: PAI-1≤4.24, 4.24<PAI-1≤5.74, 5.74<PAI-1≤6.65, 6.65<PAI-1≤7.5, 7.5<PAI-1≤8.67, 8.67<PAI-1≤10.19, 10.19<PAI-1≤12.79, 12.79<PAI-1≤17.805, 17.805<PAI-1≤20, PAI-1>20; gPlatelet count 10 quintiles: PLT≤186, 186<PLT≤206, 206<PLT≤222.5, 222.5<PLT≤237, 237<PLT≤250.5, 250.5<PLT≤265, 265<PLT≤277.5, 277.5<PLT≤301.5, 301.5<PLT≤324, PLT>324;  hprothrombin time 10 quintiles: PT≤10.5, 10.5<PT≤11, 11<PT≤11.2, 11.2<PT≤11.4, 11.4<PT≤11.6, 11.6<PT≤11.8, 11.8<PT≤11.9, 11.9<PT≤12.3, 12.3<PT≤12.6, PT>12.6; iactivated partial thromboplastin time: aPTT≤26.9, 26.9<aPTT≤28.6, 28.6<aPTT≤29.7, 29.7<aPTT≤30.85, 30.85<aPTT≤32.025, 32.025<aPTT≤33.2, 33.2<aPTT≤34.5, 34.5<aPTT≤36.05, 36.05<aPTT≤37.55, aPTT>37.55 jBUN: BUN≤0.6, 0.6<BUN≤0.6, 0.6<BUN≤0.7, 0.7<BUN≤0.7, 0.7<BUN≤0.7, 0.7<BUN≤0.7, 0.7<BUN≤0.8, 0.8<BUN≤0.8, 0.8<BUN≤0.9, BUN>0.9; kcr: cr≤80, 80<cr≤83, 83<cr≤87, 87<cr≤90, 90<cr≤94, 94<cr≤97, 97<cr≤98, 98<cr≤102, 102<cr≤112, cr>112; lFBS: FBS≤32.6, 32.6<FBS≤35.1, 35.1<FBS≤36.1, 36.1<FBS≤36.8, 36.8<FBS≤37.5, 37.5<FBS≤38.3, 38.3<FBS≤39.1, 39.1<FBS≤39.9, 39.9<FBS≤41.4, FBS>41.4; mHct: Hct≤37.8, 37.8<Hct≤46.9, 46.9<Hct≤52.6, 52.6<Hct≤57.3, 57.3<Hct≤59.4, 59.4<Hct≤62.1, 62.1<Hct≤66.3, 66.3<Hct≤76.1, 76.1<Hct≤87.4, Hct>87.4; nHDL: HDL≤50, 50<HDL≤60, 60<HDL≤71, 71<HDL≤90, 90<HDL≤109, 109<HDL≤138, 138<HDL≤211, 211<HDL≤279, 279<HDL≤401, HDL>401; oTG: TG≤2.62, 2.62<TG≤4.44, 4.44<TG≤5.01, 5.01<TG≤5.55, 5.55<TG≤6.04, 6.04<TG≤6.42, 6.42<TG≤7.35, 7.35<TG≤8.16, 8.16<TG≤9.98, TG>9.98; pFSH: FSH≤1.88, 1.88<FSH≤2.92, 2.92<FSH≤3.34, 3.34<FSH≤3.88, 3.88<FSH≤4.55, 4.55<FSH≤5, 5<FSH≤5.74, 5.74<FSH≤7.04, 7.04<FSH≤9.8, FSH>9.8; qLH: LH≤11, 11<LH≤16.3, 16.3<LH≤21.5, 21.5<LH≤25.7, 25.7<LH≤29.85, 29.85<LH≤36, 36<LH≤40.3, 40.3<LH≤46.5, 46.5<LH≤61.2, LH>61.2; rE2: E2≤9, 9<E2≤11, 11<E2≤13, 13<E2≤15, 15<E2≤17, 17<E2≤19.5, 19.5<E2≤21.5, 21.5<E2≤25, 25<E2≤30, E2>30; sProlactin: Prolactin≤6.42, 6.42<Prolactin≤7.44, 7.44<Prolactin≤8.72, 8.72<Prolactin≤10.22, 10.22<Prolactin≤11.83, 11.83<Prolactin≤13.9, 13.9<Prolactin≤15.66, 15.66<Prolactin≤19.36, 19.36<Prolactin≤30.91, Prolactin>30.91; | | | | | | | | | | | | | | | | | | | | | | | | | | | | | | |
