# Supplementary material for: 3′-UTR Polymorphisms in the Vascular Endothelial Growth Factor Gene (VEGF) Contribute to Susceptibility to Recurrent Pregnancy Loss (RPL)
Source: Int J Mol Sci. 2019 Jul 5;20(13):3319. doi: 10.3390/ijms20133319 (PMC6651559; doi:10.3390/ijms20133319)
Supplement: Supplementary file 1 [file ijms-20-03319-s001.zip › ijms-491329-suppl/ijms-491329-suppl-fig.zip!/Suppl Figure - VEGF 3-UTR in RPL (IJMS-2019-3-19).docx]

miR-200b

(2984)

miR-150,339-5p,486-5p

(500,526,730)

**Supplementary Figure 1**. miRNA-target sites in the human 3’-UTR of *VEGF* mRNA. Position of miRNA target site is numbered associated

with *VEGF* mRNA sequence.

3’-UTR of *VEGF* mRNA

VEGF 3’-UTR Validation

ORF

500

1000

1500

2000

3000

rs3025040

rs10434

(911)

rs3025053

(1024)

miR-199a-5p

miR-199b-5p

(750)

miR-107,103,125a-3p

(800,825,875)

miR-140-5P,342-3P,505,339-5P,200a,

(1060,1120,1125,1150,1220)

miR-613,300,329,23a,410

(1510,1570,1580,1600,1651)

miR-297,299,

(2023,2043)

miR-495,29a,101,300,383

(1726,1730,1735,1880,1922)

**Supplementary Figure 2. Linkage disequilibrium (LD) patterns of VEGF SNPs.**

Values in squares are LD between single markers. There were strong LDs between loci 1451C>T (3025040) and 1612G>A (rs10434, D’=0.872,LOD=6.35, r-squared=0.032), 1451C>T (3025040) and 1725G>A (rs3025053, D’=1.000,LOD=4.25,r-squared=0.014), 1612G>A (rs410434) and 1725G>A(rs3025053, D’=0.887,LOD=32.93,r-squared=0.251) in RPL subjects.

|  | FSH > 7.53 mIU/mL | FSH ≤ 7.53 mIU/mL |
| --- | --- | --- |
| VEGF 1451 C>T CC | 1 | 2.238 |
| VEGF 1451 C>T CT+TT | 1.283 | 4.635 |

**Supplementary Figure 3. RPL(recurrent pregnancy loss) incidence by interactions with FSH .We suggest that the FSH may be risk factor, associated with increase risk of RPL in VEGF 1451 C>T polymorphisms.**
